# Effectiveness of GeneXpert^®^ in the diagnosis of tuberculosis in people living with HIV/AIDS

**DOI:** 10.11606/s1518-8787.2021055003125

**Published:** 2021-12-01

**Authors:** Mariana Gaspar Botelho Funari de Faria, Rubia Laine de Paula Andrade, Ana Julia Gonçalves Camillo, Karina Fonseca de Souza Leite, Nanci Michele Saita, Valdes Roberto Bollela, Carlos Eduardo Menezes de Rezende, Aline Aparecida Monroe

**Affiliations:** I Universidade de São Paulo Escola de Enfermagem de Ribeirão Preto Programa de Pós-Graduação em Saúde Pública Ribeirão Preto SP Brasil Universidade de São Paulo. Escola de Enfermagem de Ribeirão Preto. Programa de Pós-Graduação em Saúde Pública. Ribeirão Preto, SP, Brasil; II Universidade de São Paulo Escola de Enfermagem de Ribeirão Preto Departamento Materno-Infantil e Saúde Pública Ribeirão Preto SP Brasil Universidade de São Paulo. Escola de Enfermagem de Ribeirão Preto. Departamento Materno-Infantil e Saúde Pública. Ribeirão Preto, SP, Brasil; III Universidade de São Paulo Faculdade de Medicina de Ribeirão Preto Departamento de Clínica Médica Ribeirão Preto SP Brasil Universidade de São Paulo. Faculdade de Medicina de Ribeirão Preto. Departamento de Clínica Médica e Divisão de Moléstias Infecciosas. Ribeirão Preto, SP, Brasil; IV Ministério da Saúde Agência Nacional de Saúde Suplementar Rio de Janeiro RJ Brasil Ministério da Saúde. Agência Nacional de Saúde Suplementar. Rio de Janeiro, RJ, Brasil

**Keywords:** Tuberculosis, Pulmonary, diagnosis, Molecular Diagnostic Techniques, HIV Infections, AIDS-Related Opportunistic Infections

## Abstract

**OBJECTIVE:**

To identify and synthesize the scientific knowledge produced regarding the effectiveness of the GeneXpert test in the diagnosis of pulmonary tuberculosis (TB) in people living with HIV/AIDS.

**METHODS:**

Integrative literature review, which was searched on Embase, Scopus, PubMed, Cinahl, Academic Search Premier, Socindex, and LILACS platforms, in December 2019. The studies surveyed went through two stages of selection: reading of titles and abstracts by two reviewers independently; using the Rayyan platform and reading. Nineteen primary studies in English, Portuguese, and Spanish that answered the study’s guiding question were included: How effective is the GeneXpert test in the diagnosis of pulmonary TB in people living with HIV/AIDS?

**RESULTS:**

The use of GeneXpert substantially increased the detection of TB cases among the population co-infected with HIV/AIDS, with sensitivity ranging from 68% to 100%, superior to sputum smear microscopy. Specificity ranged from 91.7% to 100%; the positive predictive value from 79.2% to 96.1%; and the negative predictive value from 84.6% to 99.3%. These values were considered similar to sputum smear microscopy by most studies. We also compared these results with different ways of performing culture and other molecular tests, being considered inferior only to the Xpert Ultra.

**CONCLUSION:**

It is possible to affirm that places with a high incidence of HIV/AIDS would benefit from the implementation of the GeneXpert test, entailing effectiveness in diagnosing pulmonary TB in this population when compared to sputum smear microscopy, a widely used test for detection of cases.

## INTRODUCTION

Since the 1980s, infection by the human immunodeficiency virus (HIV) has reinforced the concern with tuberculosis (TB)^1^. In 2015, according to the World Health Organization (WHO), 10.4 million people developed TB; 1.2 million corresponded to people with HIV/AIDS^2^.

In the 1990s, TB control strategies were based on passive detection of the disease through sputum smear microscopy in patients with chronic cough. However, the clinical presentation of pulmonary TB among people living with HIV/AIDS is nonspecific in advanced stages, with less frequent coughing and negative sputum smears^3^.

Although this microbiological diagnostic technique offers advantages in terms of costs and simplicity, specificity and sensitivity are still considered precarious, especially among people living with HIV/AIDS^4,5^, given the scarce production of sputum and the decrease in the bacillary load^6^. In addition to microbiological results, the diagnosis of TB must be supported by the clinical presentation^7^ and radiographic findings^8^.

Another microbiological test used for the diagnosis of TB, considered the gold standard for laboratory confirmation of the disease, is the sputum culture. This test has high sensitivity in detecting *Mycobacterium tuberculosis* (MTB) and has been recommended for people with HIV/AIDS^9^. However, performing such examination requires resources, qualified technical skills, and time to obtain results, which delays the diagnosis and increases the risk of disease transmission^10,11^.

So the diagnosis of TB in people living with HIV/AIDS was often based on the clinical presentation, considering the lack of microbiological proof of infections^12^. This led to the expectation of developing new diagnostic means for use by health services, among which the rapid molecular tests (TRM)^13^, such as the GeneXpert^®^ MTB/RIF (GeneXpert). This is a nucleic acid amplification-based test, based on polymerase chain reaction (PCR) whereby it detects MTB and its resistance to rifampicin in a sputum sample within a period of two hours^14^.

This test was approved in 2010 by the WHO, which recommended its use for the initial diagnosis of TB and suspected cases of multidrug-resistant tuberculosis (MDR-TB) in sputum samples^15^, and expanded this recommendation in 2013, also for the diagnosis of TB in non-respiratory samples, that is, for extrapulmonary TB^13^. In 2014, as a result of these recommendations, 3,269 GeneXpert devices were made available to public sectors in 108 of the 145 countries eligible to obtain the equipment^7^.

This review intends to identify and synthesize scientific knowledge about the effectiveness of the GeneXpert test in the diagnosis of pulmonary TB in people living with HIV/AIDS.

## METHODS

This is an integrative review, which has the potential to gather information on a given topic and present the state of the art of the object of study. We also intend to guide the definition of concepts, identify gaps, and review theories and methodological analysis, besides informing and assist practices and policy initiatives related to the issue^17^.

We took the following steps: elaboration of the study question; bibliographical survey and selection of primary studies; extracting information from selected studies; assessment of the methodological quality of the included studies; synthesis and discussion of the results found. This review is guided by the following question: How effective is the GeneXpert test in the diagnosis of pulmonary TB in people living with HIV/AIDS?

This question allowed the identification of descriptors, using the PICO strategy, proposed by The Joanna Briggs Institute (2017)^18^ and presented in Box 1.

**Box 1:** Descriptors derived from the study question, according to the PICO strategy.

Acronym	Definition	Descriptors (controlled vocabulary in bold)
P	Population	People living with **HIV/ AIDS**
I	Intervention	Carrying out the GeneXpert^®^ MTB/RIF^a^ test on sputum samples
C	Control or comparator^b^	Comparison with other diagnostic tests
O	*(outcome)*	Effectiveness in the diagnosis of pulmonary **tuberculosis**

^a^ No controlled vocabulary was found for the term in question, but the term and its derivations were used in the search.

^b^ They were not used as descriptors in the search.

Source: authors (2019).

The descriptors in bold, mentioned in Box 1, are part of the controlled vocabulary consulted in the Health Sciences Descriptors (DECS), whereby it was also possible to identify the corresponding terms in Spanish and English. For terms in English, we also consulted the Medical Subject Headings (MeSH). Finally, the free vocabulary used in the writing of publications was also sought, searching for synonyms in DECS and MeSH and databases selected for the study.

We carried bibliographic survey out in December 2019 using the vocabulary found (Box 2) and the Boolean operators OR (among words with the same meaning) and AND (among groups of words with the same meaning).

**Box 2:** Vocabulary used to search for articles for an integrative review on the effectiveness of the GeneXpert® MTB/R test in the diagnosis of pulmonary tuberculosis in people living with HIV/AIDS.

Word Groupe	Controlled vocabulary/open vocabulary
G1	**HIV** OR Síndrome de Imunodeficiência Adquirida OR aids OR HIV OR Síndrome da Deficiência Imunológica Adquirida OR Síndrome de Deficiência Imunológica Adquirida OR Síndrome da Imunodeficiência Adquirida OR Vírus da Imunodeficiência Humana OR Vírus de Imunodeficiência Humana *(Português)* **VHI** OR “Acquired Immunodeficiency Syndrome” OR AIDS OR “Human Immunodeficiency Virus” OR “Immune Deficiency Syndrome” OR “Immuno Deficiency Syndrome” OR “Immuno-Deficiency Syndrome” OR “Immuno-Deficiency Syndromes” OR “Immunodeficiency Syndromes” OR “Immunologic Deficiency Syndrome” OR “Acquired Immunodeficiency”(*Inglês)* **SIDA** OR “Síndrome de Inmunodeficiencia Adquirida” OR “Síndrome de Deficiencia Inmunológica Adquirida” OR “Síndrome de la Inmunodeficiencia Adquirida” OR VIH OR “Virus de Inmunodeficiencia Humana” OR “Virus de la Inmunodeficiencia Humana” OR “Virus del SIDA” (*Espanhol)*
G2	**Tuberculose** OR TB *(Português)* **Tuberculosis** OR TB*(Inglês)* **Tuberculosis** OR TB *(Espanhol)*
G3	GeneXpert OR “teste rápido molecular” OR TRM OR “teste molecular rápido” *(Português)* Xpert OR GeneXpert OR Cepheid OR “rapid molecular test” *(Inglês)* “prueba rápida molecular” OR PRM OR “prueba molecular rápida”*(Espanhol)*

Source: authors (2019).

CINAHL: Cumulative Index to Nursing and Allied Health Literature; ASP: Academic Search Premier; SocINDEX: bibliographic database for sociology research; LILACS: *Literatura Latino-Americana e do Caribe em Ciências da Saúde*; PubMed: Public/Medline ou Publisher Medline; SCOPUS: SciVerse Scopus Elsevier’s property; EMBASE: Excerpta Medica dataBASE.

The bibliographic survey was executed in December 2019, in the following databases: Excerpta Medica dataBASE (EMBASE), SciVerse Scopus (SCOPUS) on behalf of Elsevier, Public/Medline or Publisher Medlin (PubMed), Cumulative Index to Nursing and Allied Health Literature (CINAHL), Academic Search Premier (ASP), Bibliographic Database for Sociology Research (SocINDEX) and *Literatura Latino-Americana e do Caribe em Ciências da Saúde* (LILACS). For the LILACS search, we used the vocabulary in the three mentioned languages; in other databases, the English vocabulary was used. It is important to emphasize that we did not use publication year nor language limits in the bibliographic survey.

This search resulted in the identification of 1,802 publications, which were exported to the systematic review application Rayyan QCRI from Qatar Computing Research Institute^19^, which identified and allowed the exclusion of 597 duplicate publications. Two independent reviewers judged the eligibility of the remaining publications by reading their titles and abstracts. The following criteria guided the inclusion of 19 studies in this review: primary studies; having people with TB-HIV co-infection as the study population; address the diagnosis of pulmonary TB; and studies that answered the guiding questions of this review. We also highlight that all studies considered in this review were only based on the GeneXpert technology produced by the company Cepheid. Articles that studied cost-effectiveness, abstracts published in annals, and articles that did not compare the GeneXpert test with other diagnostic tests were excluded (Figure).

**Figure:**
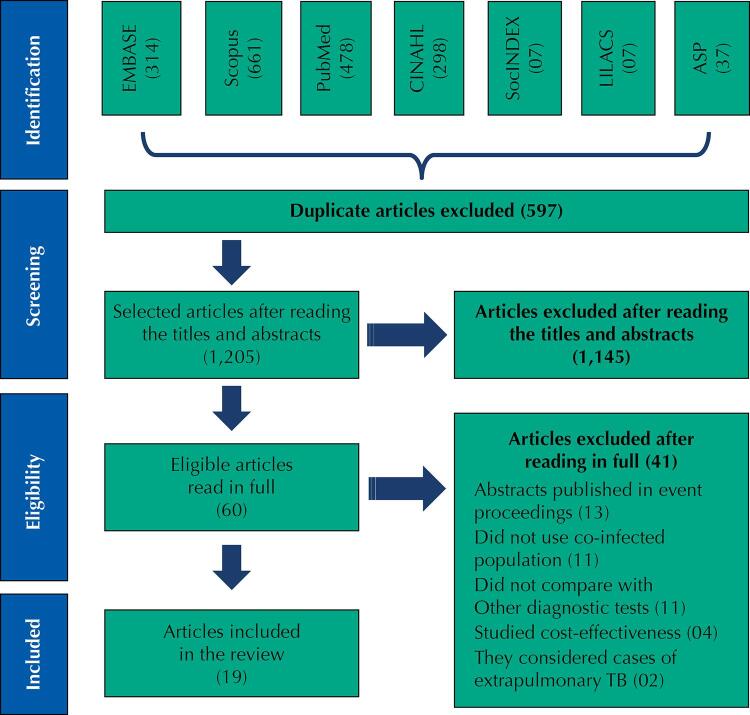
Description of articles included in the integrative review on the effectiveness of the GeneXpert® MTB/RIF test in the diagnosis of pulmonary TB in people living with HIV/AIDS.

Source: Moher D et al. (2009) adaptation^20^.

Data from the articles included in this review were extracted using a specific instrument based on Ursi (2005)^21^, allowing the narrative synthesis of its results. Finally, the methodological quality of the articles was assessed using the “Checklist for Studies on the Accuracy of Diagnostic Tests”, proposed by The Joanna Briggs Institute (JBI) (https://joannabriggs.org/critical-appraisal-tools). It includes ten evaluation items within dichotomous answers (yes/no), considering “yes” a positive answer. Two checklist items were not applicable to the selected studies, so we sorted them out during evaluations.

## RESULTS

From the 19 articles included in this review, 18 (94.7%) were published in English^6,22^ and one (5.3%) in Spanish^39^. 15 of them (78.9%) were from Africa ^6,22,23,25^, two (10.5%) from the American Continent^37,39^, one (5.3%), from Asia^24^ and one (5.3%) from a multicenter study^35^ that involves countries from Africa, America, Europe, and Asia.

The articles were published in 2011 (21,1%)^6,23,25,30^, 2012 (15,8%)^29,37,38^, 2014 (15,8%)^26,32,36^, 2015 (5,3%)^22^, 2016 (5,3%)^33^, 2017(15,8%)^24,28,34^ and 2018 (21,1%)^27,31,35,39^. Regarding the investigation time, four (21.1%) studies lasted up to 6 months^22^, five (26.3%), from 6 months to one year^6,32,35,36,39^,eight (42.1%), from one to two years^25,26,28,29,31,33,37,38^ and two (10.5%), from 2 to 3 years^27,30^.

The summary of the main results on the effectiveness of the GeneXpert is presented in Box 3. Eleven studied^6,23,25^ the sensitivity of the test, nine^6,23,25^ evaluated the specificity and eight, the positive predictive value (PPV) and the negative predictive value (NPV). The comparison tests used to assess the effectiveness of the GeneXpert test were: 14 (73.6%) sputum smear microscopy^6,23^, four (21.1%) culture in Lowenstein-Jensen (LJ)^32,34,35,39^, 13 (68.4%) culture in liquid medium ^6,22,23,25^, one (5.3%) Light Cycler Mycobacterium Detection (LCTB)^6^ and one (5.3%) MTBDRplus (Hain Lifescience)^6^. Three articles^30,32,33^evaluated effectiveness of the test according to the results of CD4+ T lymphocyte counts and one article^33^ evaluated this effectiveness according to the time of sputum collection.

**Box 3:** Description of the articles included in the integrative review on the effectiveness of the GeneXpert® MTB/RIF test in the diagnosis of pulmonary TB in people living with HIV/AIDS.

Authors Periodic Year/country	Study design	Objectives	Total (n)	Measuring instrument	Achievements	Highly rated items/ Number of Items (Limitations)
SCOTT, L. et al.^6^/ PLOS Medicine/ 2011/ South Africa	Prospective study.	Evaluate the performance of the Xpert MTB/RIF test, LightCycler Mycobacterium Detection (LCTB) and MTBDRplus (Hain Lifescience) test with smear microscopy and liquid culture in a single sputum sample.	311 individuals from a population with Prevalence of HIV.	GeneXpert MTB/RIF, Ziehl-Neelsen smear microscopy, MGIT culture, MDRTBplus and LCTB.	GeneXpert – sensitivity among people with HIV 84% (95%CI 69–93), specificity 96% (95%CI 89–99), PPV 92% (95%CI 79–98), NPV 92% (95%CI 84–97). Smear microscopy – sensitivity 54% (95%CI 38–69), specificity 100% (95%CI 95–100), PPV 100% (95%CI 85–100), NPV 80% (95%CI 70-87). MDRTBplus – 70% sensitivity (95%CI 54–83), specificity 96% (95%CI 89–99), PPV 91% (95%CI 76–98), NPV 85% (95%CI 76–92). LCTB – 70% sensitivity (95%CI 54–83), specificity 98% (95%CI 93–100), PPV 97% (95%CI 83–100), NPV 86% (95%CI 77–92).	6/8(2N/A) - it is not possible to know if the results of the reference test (sputum culture) were obtained without knowing Xpert results; - the study had 22.6% sample loss.
Dagnra et al.^22^/ New Microbes and New Infections/ 2015/Togo	Did not quoted.	Determine the prevalence of MDR-TB among previously treated HIV positive and negative patients and evaluate the performance of GeneXpert MTB/RIF in detecting MDR cases.	42 sputum samples.	GeneXpert MTB/RIF, BACTEC MGIT 960 culture, and Ziehl-Neelsen staining microscopy.	Only 31 (73.8%) of the 42 evaluated cases consented to be tested for HIV. 07 (22.6%) were HIV positive. All cases of coinfection were diagnosed by GeneXpert and two tested positive for resistance to rifampicin. There was excellent agreement between the sensitivity test and GeneXpert in the two people with MDR-TB.	6/8 (2 N/A) - not elucidated sampling process and reduced sample size, mainly regarding the number of co-infected; - it is not possible to know if the results of the reference test (sputum culture) were obtained without knowing GeneXpert results.
Rachow et al.^23^/ PLOS ONE/ 2011/ Tanzania	Clinical validation study.	Compare GeneXpert results to microscopy and culture in sputum samples from people suspected to have TB and to be infected or not with HIV.	292 patients, and 876 sputum samples.	GeneXpert MTB/RIF, Ziehl-Neelsen staining microscopy and culture in LJ and BACTEC MGIT 960 methods.	Among 77 individuals undergoing empirical treatment for TB (smear microscopy and negative culture), 07 were positive for the GeneXpert test. They were living with HIV. Sensitivity of the GeneXpert test in individuals with HIV: 82.0% (95%CI 68.6–91.4), smear microscopy: 52.0% (95%CI 37.4–66.3), culture (LJ): 68.0% (95%CI 53.3–80.5), and (MGIT): 76.0% (95%CI 61.8–86.9). Sensitivity of both smear microscopy and culture (LJ): 78.0% (95%CI 64.0–88.5). Specificity for the GeneXpert test was 98.0% (95%CI 89.4–100). Regarding the other tests, the specificity was 100% (95%CI 92.9–100).	8/8 (2 N/A**).**
Sumangala et al.^24^/ Journal of International Medicine & Dentistry/ 2017/ India	Cross-sectional study.	Assessing the Effectiveness of the GeneXpert MTB/RIF Assay in the Early Diagnosis of Tuberculosis in People Living with HIV.	433 individuals and sputum samples.	GeneXpert MTB/RIF and Fluorescent Microscopy.	Among 433 sputum samples, 36 (8.3%) were positive for GeneXpert and 28 (6.4%) samples were positive for fluorescent microscopy. Five cases of resistance to rifampicin were detected by GeneXpert.	5/8 (2N/A) - it is not possible to know if the results of the reference test (sputum culture) were obtained without knowing GeneXpert results; - used sputum smear microscopy as the reference diagnostic method, having limitations to correctly classify a case of the disease.
Lawn1et al.^25^/ PLOS Medicine/ 2011/ África do Sul	Prospective study.	Evaluate the diagnostic accuracy of the GeneXpert MTB/RIF test in the diagnosis of TB in patients with advanced immunodeficiency and who have not started ART.	445 individuals and 839 sputum samples.	GeneXpert MTB/RIF, microscopy, liquid culture by BACTEC MGIT 960.	Among 445 subjects participating in the study, 81 had a positive result for the culture. GeneXpert – Sensitivity 73.3% (95%CI 61.9–82.9); specificity 99.2% (95%CI 97.7–99.8); PPV 94.8% (95%CI 85.6-98.9); NPV 94.8% (95%CI 92.1–96.8). GeneXpert had a low false-positive rate and detected all positive TB cases for both smear microscopy and culture tests. Sputum smear microscopy – Sensitivity 28.0% (95%CI 18.2–39.6); specificity 100.0% (95%CI 98.9–100.0); 100% PPV (95%CI 83.9–100); NPV 87.3% (95%CI 83.7–90.3). GeneXpert detected 43.4% of smear-negative and culture-positive cases in a single sputum sample and 62.3% when two sputum samples were analyzed. The test correctly identified rifampicin resistance in four patients who had MDR-TB, rendering three false positive drug resistance results.	8/8 (2N/A).
Balcha et al.^26^/ PLOS ONE/ 2014/ Ethiopia	Did not quoted.	Evaluate the prevalence of TB, the diagnostic yield of GeneXpert MTB/RIF compared to sputum smear microscopy and culture, and the impact of GeneXpert results on the clinical treatment of HIV-positive adults eligible for ART.	812 individuals and 1,514 sputum samples.	GeneXpert MTB/RIF, Ziehl-Neelsen stained microscopy of smear and liquid culture.	Among 137 bacteriologically confirmed TB patients, 123 (89.8%) were culture positive and 13 (10.2%) were negative. The GeneXpert test was positive for 94 individuals, which also detected 2 cases of resistance to rifampicin. The sputum smear microscopy was positive for 31 patients. Regarding smear microscopy, GeneXpert increased the detection of TB cases by 47.4% (64 cases). Moreover, GeneXpert detected 14 (10.2%) cases of culture-negative or contaminated TB. Among 94 patients with sputum samples positive for GeneXpert, 30 (32%) were positive for sputum smear microscopy. Of the 122 culture-positive cases, 28 (23%) were smear-positive. Of 104 smear-negative cases, 54 (52%) were positive using both GeneXpert and culture, and 40 (38%) were positive using culture alone. All patients with positive smear microscopy were positive for GeneXpert or culture. Moreover, 81 [66.4% (95%CI 57.2–74.6)] culture-positive TB cases were positive for GeneXpert. GeneXpert – sensitivity 96.4% (95%CI 79.7–99.9) and 57.4% (95%CI 46.8–67.5) between positive and negative sputum smear microscopy cases, respectively; specificity 98.4% (95%CI 97.0–99.2); PPV 88.0% (95%CI 79.2–93.6) and NPV 94.2% (95%CI 92.1–95.8). Smear microscopy – 23% sensitivity (95%CI 16.0–31.7).	8/8 (2N/A).
Griesel et al.^27^/ Clinical Infectious Diseases/ 2018/ South Africa	Prospective cohort study.	Develop a clinical prediction rule for the diagnosis of tuberculosis and determine the diagnostic utility of the GeneXpert MTB/RIF assay in critically ill HIV-infected patients.	484 individuals.	GeneXpert MTB/RIF, BACTEC MGIT 960 Culture and Fluorescence Smear Microscopy.	Considering the sputum culture, 53% (255/484) had a positive result. Among the participants, 56 (11.6%) underwent empirical treatment with negative sputum. Smear microscopy and GeneXpert, whence 22 (43.1%) had a positive result for the culture. GeneXpert – sensitivity 86.3% (95%CI 81.5–90.3); specificity 96.1% (95%CI 92.6–98.2); PPV 96.1% (95%CI 92.7-98.2); NPV 86.2% (95%CI 81.4–90.2); PLR 21.9 (95%CI 11.5–41.6); NLR 0.14 (95%CI 0.11–0.19). Sputum smear microscopy – sensitivity 57.0% (95%CI 50.7–63.2); specificity 98.7% (95%CI 96.2–97.7); PPV 98.0% (95%CI 94.2–99.6); NPV 67.2% (95%CI 61.9–72.2); PLR 43.3 (95%CI 14.0-134.0); NLR 0.44 (95%CI 0.38–0.50).	7/8(2N/A) - it is not possible to know if the results of the reference test (sputum culture) were obtained without knowing GeneXpert results.
Akanbi et al.^28^/ BMC Pulmonary Medicine/ 2017/Nigeria	Prospective cohort study.	Evaluate the impact of GeneXpert MTB/RIF on diagnosis, treatment time, and treatment outcome among HIV-associated TB patients in Nigeria.	310 individuals and 620 sputum samples.	GeneXpert MTB/RIF and smear microscopy.	TB was diagnosed in 76 (24.5%) patients, of which 63 (82.9%) had bacteriological confirmation, 56 (73.7%) had a positive result for GeneXpert and 57 (75%) for sputum smear microscopy. In the concordance analysis, 50 patients (65.8%) were positive for both tests, 6 (7.9%) were positive for GeneXpert and negative for sputum smear microscopy, 7 (9.2) negative for GeneXpert and positive for sputum smear microscopy and 13 (17.1%) were negative for both exams.	5/8(N/A) - it is not possible to know if the results of the reference test (sputum culture) were obtained without knowing GeneXpert results; - used sputum smear microscopy as a diagnostic method of comparison, which may not correctly classify a case of the disease.
O’Grady et al.^29^/ Clinical Infectious Diseases/ 2012/ Zambia	Descriptive, prospective study.	Evaluate the performance of the GeneXpert MTB/RIF Assay for the detection of pulmonary tuberculosis and MDR-TB in HIV-infected and non-HIV-infected adult patients.	643 individuals.	GeneXpert MTB/RIF, smear microscopy, BACTEC MGIT 960 culture, and MGIT drug sensitivity test.	GeneXpert in HIV-positive patients – sensitivity 88.2% (95%CI 81.9–92.6); specificity 95.1% (95%CI 1.5–97.3); PPV 92.2% (95%CI 86.5-95.7); NPV 92.5% (95%CI 88.4-95.3); sensitivity when sputum smear microscopy was positive: 96.5% (95%CI 89.3–99.1); sensitivity when sputum smear microscopy was negative: 78.9% (95%CI 67.8–87.1). GeneXpert in HIV negatives – sensitivity 74.3% (95%CI 56.4–87.0); specificity 96.3% (95%CI 91.7–98.5); PPV 81.3% (95%CI 63.0-92.1); NPV 94.5% (95%CI 89.5–97.3).	6/8(2N/A) - it is not possible to know if the results of the reference test (sputum culture) were obtained without knowing GeneXpert results; - patients with contaminated and insufficient samples and patients with negative cultures in treatment were excluded (which represents a loss of 33.4% of eligible cases).
Theron et al.^30^/ American Journal of Respiratory and Critical Care Medicine/ 2011/ South Africa	Did not quoted.	Validate GeneXpert MTB/RIF test performance using a single cartridge and assess the impact of certain factors on test performance.	480 sputum samples taken from a high HIV prevalence population.	GeneXpert MTB/RIF, fluorescence microscopy and BACTEC MGIT 960 culture.	GeneXpert in co-infected patients – 69.6% sensitivity (95%CI 55.2–80.1); sensitivity in people with negative sputum smear microscopy 47.3% (95%CI 29.2–67); specificity 91.7% (95%CI 83.8-95.9); NPV 84.6% (95%CI 75.9–90.6); PPV 82.1% (95%CI 67.3–91). GeneXpert in non-coinfected patients – sensitivity 82.9% (95%CI 73.4–89.6); specificity 95.6 (95%CI 91.8–97.7); NPV 93.3% (95%CI 89.1–96.0); PPV 88.3 (95%CI 79.3–93.4). Sputum smear microscopy in co-infected patients – sensitivity 50% (95%CI 36.1–63.9); specificity 98.9% (95%CI 94.6–99.8). In patients with HIV and a CD4 count < 200 cells/ml, the combination of sputum smear microscopy and GeneXpert had a sensitivity of 69.6% (95%CI 49.3–84.4) and isolated microscopy of 39.1% (CI95 % 22.2–59.2).	7/8(2N/A) - it is not possible to know if the results of the reference test (sputum culture) were obtained without knowing GeneXpert results.
Mbu et al.^31^/ PLOS ONE/ 2018/ Cameroon	Did not quoted.	Assess the burden of TB among people newly diagnosed with HIV and compare the performance of the WHO-recommended clinical screening algorithm and the methods of diagnosing TB in this population.	940 individuals.	GeneXpert MTB/RIF, fluorescence microscopy and culture in LJ medium and BACTEC MGIT 960.	In the 131 samples with positive results for culture, the GeneXpert test was performed retrospectively in 108 (82%) of them. GeneXpert – 68% sensitivity (95%CI 58–76); sensitivity in people with positive sputum smear microscopy 97%; sensitivity in people with negative sputum smear microscopy 51%. Smear microscopy – sensitivity of direct smear microscopy 24% (95%CI 18–32); sensitivity of concentrated sputum smear microscopy 35% (95%CI 28–44). Culture: sensitivity – solid 81% (95%CI 73–87); net 98% (95%CI 94–99).	6/8(2N/A) - the results of the reference test (sputum culture) were obtained before the knowledge of the Xpert results; - the GeneXpert test was only performed on culture-positive samples, resulting in a significant sample loss.
Ssengooba et al.^32^/ PLOS ONE/ 2014/ Uganda	Prospective study.	Compare the diagnostic gain of several complementary GeneXpert strategies with that of an Xpert replacement strategy for the diagnosis of pulmonary TB among HIV-infected adults.	424 individuals.	GeneXpert MTB/RIF, smear microscopy and culture in solid and liquid media (MGIT and LJ).	123 (29.0%) participants tested positive for MGIT culture. Rifampicin resistance – 04 positive for GeneXpert and MGIT. GeneXpert – sensitivity 76.4% (95%CI 67.9–83.6); sensitivity among participants with CD4 > 200 cells/mm^3^ 91,7% (95%CI 73.0–98.9%); sensitivity among participants with CD4 ≤ 200 cells/mm^3^ 73,2% (CI 63.2–81.7%). There was almost no difference in test sensitivity between participants with CD4 of 50 to 200 cells/mm^3^ and participants with CD4 < 50 cells/mm^3^ (68.9% vs 76.9%, p = 0.491). LJ culture – sensitivity 81.3% (95%CI 73.2–87.7). Ziehl Neelsen – sensitivity 31.7% (CI 23.6–40.7). Direct fluorescence microscopy - sensitivity 35.0% (95%CI 26.5–44.0). Concentrated fluorescent microscopy – sensitivity 43.9% (95%CI 34.9–53.1).	6/8(2N/A) - sampling was neither consecutive nor random (used secondary data); - it is not possible to know if the results of the reference test (sputum culture) were obtained without knowing GeneXpert results.
Cavanaugh et al.^33^/ PLOS ONE/ 2016/ Kenya	Did not quoted.	Characterize the value of various diagnostic tests, including the incremental yield of GeneXpert MTB/RIF and the culture on sputum smear microscopy.	778 people living with HIV.	GeneXpert MTB/RIF, direct Ziehl-Neelsen smear microscopy, concentrated fluorescence microscopy, and MGIT 960 culture.	88 (11.3%) cases of TB were confirmed. GeneXpert – detected 43 cases (incremental yield = 24.4%). In specific samples, it identified 48/84 cases (57.0%) while the morning samples identified 50/76 (66.0%) cases. Two GeneXpert tests detected 22/24 (92.0%) cases of TB with CD4 < 100 cells/μL and 30/45 (67.0%) cases with CD4 counts ≥ 100 cells/μl. GeneXpert identified 45 (28.0%; 95%CI 16.6–39.3) patients with negative smears; identified six patients who were not identified by culture. Microscopy – detected 25 (33.6%) cases in 76 morning sputum samples, 24 (31.5%; 95%CI 23.4–39.6) were identified by ZN microscopy and 29 (6.5% 95%CI 2.8–10.2) by FD. Liquid culture – identified 10 (13.0%; 95%CI 6.4–19.7) additional cases that were not identified by microscopy or GeneXpert. In total, liquid culture and GeneXpert performed on a single morning sample identified 60 (79.0%; 95%CI 72.4–85) cases of pulmonary TB.	7/8(2N/A) - it is not possible to know if the results of the reference test (sputum culture) were obtained without knowing GeneXpert results.
Mollel et al.^34^/ Tanzania Journal of Health Research/ 2017/ Tanzania	Cross-sectional study.	Evaluate the performance of GeneXpert MTB/RIF in the diagnosis of TB in TB/HIV co-infected patients.	69 individuals.	GeneXpert MTB/RIF, microscopy and LJ method culture.	GeneXpert resulted in 09 positive results for TB and 60 negative results. Sensitivity – 100% (95%CI 66.4–100); specificity – 100% (95%CI 94–100); PPV 79.2% (95%CI 39–91.5); NPV 98.9% (95%CI 95–99.6). Microscopy – 05 positives, 01 false positive, 59 negatives and 04 false negatives. Sensitivity – 55.6% (95%CI 21.4–86.3); specificity – 98.3% (95%CI 91–100); PPV 75.6% (95%CI 28.9–95.9). NPV 96.0% (95%CI 92.0–98.0).	7/8(2N/A) - it is not possible to know if the results of the reference test (sputum culture) were obtained without knowing GeneXpert results.
Dorman et al.^35^/ The Lancet Infectious Diseases/ 2018/ South Africa, Uganda, Kenya, India, China, Georgia, Belarus, and Brazil	Prospective study.	Compare the diagnostic accuracy of the Xpert MTB/RIF Ultra test with that of the GeneXpert MTB/RIF for detection of negative smear tuberculosis and resistance to rifampicin.	1,753 adult individuals with symptoms of pulmonary tuberculosis.	Xpert MTB/RIF Ultra test, smear microscopy using Ziehl-Neelsen and culture MGIT BACTEC 960 and LJ.	The sensitivities of the Xpert Ultra and Xpert were 90% (95%CI 83–95) and 77% (95%CI 68–84), respectively, for the 115 HIV-positive participants with culture-positive sputum. The specificities of Xpert Ultra and Xpert for detection were respectively 96% and 98% overall and 93% and 98% for patients with a prior history of tuberculosis.	6/8(2N/A) - it is not possible to know if the results of the reference test (sputum culture) were obtained without knowing GeneXpert results; - the study had 22.6% sample loss.
Auld et al.^36^/ Public Health Action/ 2014/ Cambodia	Did not quoted.	Describe the implementation and use of the GeneXpert MTB/RIF test in the diagnosis of tuberculosis among people living with HIV/AIDS.	497 individuals.	GeneXpert MTB/RIF, Smear and Culture Microscopy.	Among the 497 people with HIV, 357 (72%) were tested with sputum smear microscopy and 250 (50%) with Xpert; of which 25 (10%) were positive for TB and none resistant to rifampicin. Only 22/357 (4%) had sputum culture, being 04 (18%) positive for *M. tuberculosis* and 06 (27%) for non-tuberculous mycobacteria (NTM).	4/8(2N/A) - sampling was neither consecutive nor random (used secondary data); - it is not possible to know if the results of the reference test (sputum culture) were obtained without knowing GeneXpert results; - used sputum smear microscopy as a diagnostic method of comparison, which may not correctly classify a case of the disease; - the reference standard test was not the same for all participants.
Balcells et al.^37^/ The International Journal of Tuberculosis and Lung Disease / 2012/ Chile	Study cross-sectional.	To assess the diagnostic accuracy of the GeneXpert MTB/RIF Assay in detecting pulmonary TB in HIV-infected patients in clinical care compared to traditional rapid smear microscopy and culture.	160 individuals.	GeneXpert MTB/RIF, smear microscopy and cultures in LJ medium and MGIT BACTEC 960.	GeneXpert – sensitivity 91.7% (95%CI 64.6–98.5); specificity 99.3% (95%CI 96.3–99.9). PPV 91.7% (95%CI 64.6-98.5); NPV 99.3% (95%CI 96.3–99.9). Among the 12 culture-confirmed TB cases, two cases of resistance to rifampicin were detected by the Xpert MTB/RIF (16.6%). Microscopy – sensitivity 66.7% (95%CI 39.1–86.2); specificity 98.6% (95%CI 95.2–99.6); PPV 80% (95%CI 49–94.3); NPV 97.3% (95%CI 93.3–99.3).	8/8 (2N/A).
Lawn et al.^38^/ Clinical Infectious Diseases/ 2012/ South Africa	Did not quoted.	Report the characteristics of patients living with HIV/AIDS with a clinical diagnosis of TB and a negative result for GeneXpert MTB/RIF and report the Xpert status for subsequent pragmatic and clinical results.	602 sputum samples.	GeneXpert MTB/RIF, Fluorescence Microscopy and Liquid Culture.	*M. tuberculosis* was cultivated in 01 sample of 89 patients, which represented a prevalence of 17.0% (95%CI 13.9–20.5) of TB. Among them, 24 (27.0%) cases were positive for sputum smear microscopy and 52 (58.4%) for GeneXpert. When 02 samples were collected, the positivity for GeneXpert was 64 (71.9%).	8/8 (2N/A).
Cuervo et al.^39^/ Venezolan Archives of Pharmacology and Therapeutics/ 2018/Cuba	Study retrospective.	To reaffirm the impact-benefit of using GeneXpert MTB/RIF for the diagnosis of tuberculosis in HIV/AIDS patients with suspected pulmonary TB.	152 sputum samples.	GeneXpert MTB/RIF, smear microscopy in Ziehl-Neelsen medium and culture by LJ.	Of the 152 samples, GeneXpert detected 39 (25.65%) positive for *Mycobacterium Tuberculosis*, 36 (23.68%) being non-resistant to rifampicin, and 03 (2.00%) resistant to rifampicin. The percentage of positivity for sputum smear microscopy was 11.8% and for LJ culture was 21.7%. In comparison, researchers observed that the sensitivity, specificity, and agreement were above 90%, with no statistically significant differences between them.	5/8 (2N/A) - study is retrospective (possibly used secondary data); - information about losses and exclusions of people from the sample was not revealed; - it is not possible to know if the results of the reference test (sputum culture) were obtained without knowing GeneXpert results.

HIV: human immunodeficiency virus; 95%CI: 95% confidence interval; LJ: Löwenstein Jensen; MDR: multidrug-resistant; N/A: not applicable; WHO: World Health Organization; PLR: positive likelihood ratio; NLR: negative likelihood ratio; ART: antiretroviral therapy; TB: tuberculosis; PPV: positive predictive value; NPV: negative predictive value.

As for the methodological quality of the studies, five studies^23,25,26,37,38^ included all the essential items considered by The Joanna Briggs Institute. Only one (5.3%)^27^ article presented sample size calculation.

## DISCUSSION

The epidemic and the expansion of HIV/AIDS infection impacted the incidence of TB^40^, whose early detection followed by timely treatment represent the key to its control, so that laboratory diagnosis represents a challenge for health services^39^. The population living with TB/HIV co-infection has some characteristics that limit the use of diagnostic tools, as it can more easily present a negative smear, advanced immunosuppression that provides delayed diagnosis, the subclinical manifestation of signs and symptoms characteristic of TB^41^.

In TB/HIV endemic regions with limited resources, sputum microscopy is usually the only method available for diagnosis^4,32,34,42^. The use of only this technique to screen for TB in the population co-infected with HIV restricts its potential diagnosis because approximately 10,000 organisms per milliliter are needed in the sputum to perform the test, an uncommon amount among the population living with HIV/AIDS^34^.

GeneXpert test is a sensitive, specific^43^, simple, and innovative method whose objective, since its implementation, has been to detect the presence of *M. tuberculosis* and resistance to rifampicin within 2 hours^6,22^. The method has an increased screening capacity^43^, besides requiring minimal bacillary concentrations in the samples for the examination^32^.

Due to these particularities, the relevance of sputum smear microscopy for the diagnosis of TB where GeneXpert is available is questioned, although evidence found shows that the concomitant use of both tests can result in an increase in the identification of cases not detected by both an exam and on the other^28,30^, mainly in people with T CD4+ lymphocyte count < 200 cells/µl^30^.

Other studies do not address the simultaneous performance of both tests but point out that the use of GeneXpert substantially increased the detection of TB cases among the population co-infected with HIV/AIDS^24,26,33,38,42^, which was accentuated in morning sputum samples and among people with lower CD4+ T lymphocyte counts^33^.

The WHO recommendation to use GeneXpert, instead of sputum smear microscopy as the initial test of choice in HIV-infected individuals susceptible to TB^6^, corroborates the findings of studies that have shown that GeneXpert’s sensitivity in diagnosing TB in people living with HIV ranged from 68%^31^ to 100%^34^ (average 81.1%), being greater than the sensitivity of sputum smear microscopy^6,23,25^, which ranged from 23.0%^26^ to 66,7%^37^ (average 43.3%). Given the above, the implementation of GeneXpert, a fast and accurate test in different clinical contexts, has led to a significant increase in the diagnosis of pulmonary TB, both when used as a first-choice test and as a complement^45^.

We highlight that sensitivity is calculated based on the sputum culture, which is considered the gold standard test for the diagnosis of TB. Although this is a reference test, some studies compare it with GeneXpert, pointing to lesser sensitivity of the latter in relation to culture using the LJ and BACTEC MGIT 960 methods^31^, while another study shows greater^23^ sensitivity and other equal performance in relation to the LJ method^32^. Two studies brought other diagnostic methods that were also compared to GeneXpert: one indicated similar sensitivity between it and^6^ the LCTB test, and another indicated lower sensitivity when compared to XpertUltra^35^. Thus, we highlight the importance of using GeneXpert as the test of choice for the diagnosis of pulmonary TB in adults living with HIV, because it has similar and superior effectiveness compared to culture and even to other molecular methods, such as LCTB. However, it is worth point out the importance of further studies in relation to the comparison of the test with culture, because divergences came up in relation to the results regarding such effectiveness. The indication of Xpert Ultra stands out as a method of choice to replace all available tests, including GeneXpert, because only one study was found in this review that compared the two tests^46^.

It should also be noted that the sensitivity of the GeneXpert test in people living with HIV was greater than in HIV-negative people^29^, in contrast to another study that stated that the sensitivity in people living with HIV was lower^30^. The increased sensitivity of the test in HIV-positive patients adds important advantages to its use in health services, such as the increase in the detection rate of microbiologically confirmed TB and the potential to institute a rapid treatment, aiming to reduce TB transmission^47^. Some individual characteristics, such as a negative sputum smear microscopy^26,29^ and a CD4+ T lymphocyte count ≤ 200 cells/µl^32^, were mentioned as elements that reduce the sensitivity of the GeneXpert test. Although these results are controversial in relation to the study by Cavanaugh et al. (2016)^33^, which shows increased test sensitivity in people with lower CD4+ T lymphocyte counts, another study shows that the performance of GeneXpert in HIV-infected people with advanced immunosuppression has certain limitations^32^ that may be caused by the lower concentration of mycobacteria in sputum and by the occult or subclinical presentation of the disease^30^.

The specificity of GeneXpert ranged from 91.7%^30^ to 100%^34^ (average 95.6%), and in comparison with sputum smear microscopy, some studies concluded that both exams showed similar performance in relation to the mentioned aspect^6,23,27,34,37^, and others that GeneXpert has less specificity^25,30^. Regarding other diagnostic tests, the study mentions that the specificity of the GeneXpert test is similar to that of culture^23^ and LCTB^6^. The specificity of the test in people living with HIV/AIDS was also similar to people not infected by the virus^29^. Based on the above, it appears that the specificity of the GeneXpert test is comparable to other diagnostic tests.

The PPV, understood as the probability of having the disease when the test is positive, ranged from 79.2% to 96.1% (average 91%) for GeneXpert in people living with HIV/AIDS infection, is considered similar to sputum smear microscopy^6,25,27,34,37^ and to LCTB^6^, as well as when the test is performed in people who are not living with the virus^30^, although one of the studies has pointed out that the PPV was lower in these people^29^. On the other hand, NPV, understood as the probability of not having the disease when the test is negative, ranged from 84.6% to 99.3% (average 92.6%) for GeneXpert in people living with HIV/AIDS, being considered similar^34,37^ or greater^6,25,27^ in relation to sputum smear microscopy and similar to LCTB^6^ and in people who do not live with the virus^29^, although one of the studies has pointed out that the NPV is greater in these people^30^. Thus, as well as in the assessment of specificity, the results of the effectiveness of the GeneXpert test in terms of PPV and NVP showed evidence of being similar to the other comparison tests.

A study also presented the test’s likelihood ratio for diagnosing TB in co-infected patients, calculated by dividing the probability of positive (positive likelihood) or negative (negative likelihood) results, in people with the disease, by the probability of the same result in people without the disease^49^. Regarding the positive likelihood ratio, there was no difference between GeneXpert and sputum smear microscopy^27^. However, the negative likelihood ratio of GeneXpert was lower and close to “zero”^27^, favoring it if compared to sputum smear microscopy.

The effectiveness of the test in identifying cases resistant to rifampicin needs to be further studied, as one study^25^ showed that of seven identified cases, three were false positives; as well as the fact that other studies^6,22,24,32,37^ have a very small population of resistant cases, limiting the conclusions that the test has similar effectiveness to sensitivity tests^22,24,32,37^ and even to MDRTB plus^6^. So diagnosing resistance to rifampicin still remains a challenge for health services, considering it would reduce the average time to detect resistant cases to up to two days^32^, which would be an admirable performance when compared to almost 40 days of the test of sensitivity/conventional culture^25^. The immediate diagnosis of these cases would allow a substantial reduction in the risks of nosocomial transmission of the resistant bacillus and its specific and early treatment, which would result in a reduction in the severe forms of TB, as well as an improvement in the prognosis and a reduction in loss of clinical follow-up^25,39^.

The expansion of the GeneXpert test in diagnosing TB among HIV-infected individuals is essential, given its effectiveness and especially in countries with a high burden of coinfection, as this test would bring gains in case detection by replacing or complementing the sputum smear microscopy and would make the treatment time opportune^28^ in relation to the culture, which has cost limitations and technical requirements^32,33^. We also emphasize that GeneXpert’s diagnostic accuracy, based on 85% sensitivity and 97% specificity, would potentially save more than 400,000 lives per year^34^, even if some cases undergo empirical treatment, which would be reduced by locations with the implementation of testing^27,38^.

As for the methodological quality of the studies, five studies contemplated all the essential items considered by The Joanna Briggs Institute related to accuracy evaluation of tests^23,25,26,37,38^. Of the methodological limitations found in the studies, the small sample size stands out^22^; sample losses above 20%^6,29,35^; the lack of clarification regarding the sampling process^39^ and the sampling process^22^; the use of secondary data^32,36,39^; doubts about performance^6,22,27^ and nonperformance^31^ of blind studies in relation to the results of the tests performed; and the use of different reference tests for the study participants ^36^ or the use of sputum smear microscopy as a reference test^24,28,36^. Moreover, a possible population selection bias is identified in a study, within the GeneXpert test was only performed on samples that were positive for the culture^31^.

Despite the fact that the test is more costly than microscopy, the benefits of the time of diagnosis and indication of drug resistance proved to be greater, because they bring greater sensitivity and specificity. In addition, the test establishes the diagnosis in a significant proportion of smear-negative patients and has a cut-off consolidated for TB-MDR^30,45^. Based on this, GeneXpert can be used as a first-line diagnostic method^24^.

Some limitations should be noted. This review may not have included relevant studies that were not indexed in the searched databases, nor included the gray literature that could enable the identification of other topics/relevant points on the subject to be addressed.

It is possible to conclude that places with a high incidence of HIV/AIDS could benefit from the implementation of the GeneXpert test, because its effectiveness in diagnosing pulmonary TB in this population is expressive when compared to sputum smear microscopy, a test that has been used for a long time and widely used for case detection. When compared to other tests, there was controversy regarding the effectiveness of GeneXpert and the different types of cultures, similar to that of LCTB and lower than that of Xpert Ultra. In addition to increasing the detection of pulmonary TB cases among people living with HIV, the GeneXpert test can provide benefits such as faster results than those obtained by culture (the most sensitive method for confirming TB), culminating in timely detection and treatment, making GeneXpert a tool in the fight against TB. Along with the innovation in the use of GeneXpert, we stand for the support and strengthen of health services in the applicability of this technique in order to achieve the goals of sustainable development and the End TB Strategy.
