# Supplementary material for: Effectiveness of GeneXpert® in the diagnosis of tuberculosis in people living with HIV/AIDS
Source: Rev Saude Publica. 2021 Dec 1;55:89. doi: 10.11606/s1518-8787.2021055003125 (PMC8664060; doi:10.11606/s1518-8787.2021055003125)
Supplement: Translation

# Translation: Efetividade do GeneXpert^®^ no diagnóstico da tuberculose em pessoas que vivem com HIV/aids

**Authors:** Mariana Gaspar Botelho Funari de Faria, Rubia Laine de Paula Andrade, Ana Julia Gonçalves Camillo, Karina Fonseca de Souza Leite, Nanci Michele Saita, Valdes Roberto Bollela, Carlos Eduardo Menezes de Rezende, Aline Aparecida Monroe

**Affiliations:** I Brasil Universidade de São Paulo. Escola de Enfermagem de Ribeirão Preto. Programa de Pós-Graduação em Saúde Pública. Ribeirão Preto, SP, Brasil; II Brasil Universidade de São Paulo. Escola de Enfermagem de Ribeirão Preto. Departamento Materno-Infantil e Saúde Pública. Ribeirão Preto, SP, Brasil; III Brasil Universidade de São Paulo. Faculdade de Medicina de Ribeirão Preto. Departamento de Clínica Médica e Divisão de Moléstias Infecciosas. Ribeirão Preto, SP, Brasil; IV Brasil Ministério da Saúde. Agência Nacional de Saúde Suplementar. Rio de Janeiro, RJ, Brasil

**Keywords:** Tuberculose Pulmonar, diagnóstico, Técnicas de Diagnóstico Molecular, Infecções por HIV, Infecções Oportunistas Relacionadas com a AIDS

## Abstract

**OBJETIVO:**

Identificar e sintetizar o conhecimento científico produzido a respeito da efetividade do teste GeneXpert no diagnóstico da tuberculose (TB) pulmonar em pessoas vivendo com HIV/aids.

**MÉTODOS:**

Revisão integrativa da literatura, cuja busca foi feita nas plataformas Embase, Scopus, PubMed, Cinahl, *Academic Search Premier*, Socindex e Lilacs, em dezembro de 2019. Os estudos levantados passaram por duas etapas de seleção: leitura dos títulos e resumos por dois revisores de forma independente, utilizando a plataforma Rayyan e leitura integral dos mesmos. Foram incluídos 19 estudos primários em inglês, português e espanhol que respondiam à pergunta norteadora do estudo: Qual é a efetividade do teste GeneXpert no diagnóstico da TB pulmonar em pessoas que vivem com HIV/aids?

**RESULTADOS:**

A utilização do GeneXpert aumentou substancialmente a detecção de casos de TB entre a população coinfectada com HIV, com sensibilidade que variou de 68% a 100%, sendo superior à baciloscopia. A especificidade variou de 91,7% a 100%; o valor preditivo positivo, de 79,2% a 96,1%; e o valor preditivo negativo, de 84,6% a 99,3%, valores considerados semelhantes à baciloscopia pela maioria dos estudos. O teste também foi comparado com as diferentes formas de realização da cultura e outros testes moleculares, sendo considerado inferior apenas ao Xpert Ultra.

**CONCLUSÃO:**

É possível afirmar que locais com alta incidência de HIV se beneficiariam com a implantação do teste GeneXpert, uma vez que sua efetividade no diagnóstico da TB pulmonar nessa população é expressiva quando comparada à baciloscopia, teste que foi por muito tempo amplamente utilizado para a detecção dos casos.

## INTRODUÇÃO

A partir da década de 1980, a infecção pelo vírus da imunodeficiência humana (HIV) reforçou a preocupação com a tuberculose (TB)^1^. Em 2015, de acordo com a Organização Mundial da Saúde (OMS), 10,4 milhões de pessoas desenvolveram TB, das quais 1,2 milhão correspondiam às pessoas vivendo com HIV/aids^2^.

Na década de 1990, as estratégias de controle da TB partiam da detecção passiva da doença por meio de baciloscopia de escarro de pacientes com tosse crônica. No entanto, a apresentação clínica da TB pulmonar entre as pessoas que vivem com HIV/aids é inespecífica em estágios avançados, com tosse menos frequente e esfregaços de escarro negativos à baciloscopia^3^.

Embora essa técnica diagnóstica microbiológica ofereça vantagens quanto aos custos e à simplicidade, a especificidade e a sensibilidade ainda são consideradas precárias, principalmente entre as pessoas que vivem com HIV/aids^4,5^, em função da produção escassa de escarro e da diminuição da carga bacilar^6^. Dessa forma, é importante ressaltar que, além dos resultados microbiológicos, o diagnóstico de TB deve ser subsidiado pela apresentação clínica^7^ e por achados radiográficos^8^.

Outro exame microbiológico utilizado para o diagnóstico da TB, considerado padrão-ouro para confirmação laboratorial da doença, é a cultura de escarro. Tal exame tem alta sensibilidade na detecção do *Mycobacterium tuberculosis* (MTB) e seu uso foi recomendado para pessoas que vivem com HIV/aids^9^. No entanto, a realização de tal exame requer recursos e habilidades técnicas qualificadas, além de maior tempo para a obtenção do resultado, o que retarda o diagnóstico e aumenta o risco de transmissão da doença^10,11^.

Partindo dessa compreensão, muitas vezes o diagnóstico da TB em pessoas que vivem com HIV/aids se baseava unicamente na apresentação clínica, uma vez que não havia comprovação microbiológica da infecção^12^. Esse obstáculo levou à expectativa de desenvolvimento de novos meios diagnósticos para uso dos serviços de saúde, dentre os quais mereceram destaque os testes rápidos moleculares (TRM)^13^, como o GeneXpert^®^ MTB/RIF (GeneXpert), que consiste em um teste de amplificação de ácido nucleico baseado em reação em cadeia da polimerase (PCR), por meio do qual se detecta o MTB e sua resistência à rifampicina em uma amostra de escarro dentro de um período de duas horas^14^.

Esse teste foi aprovado em 2010 pela OMS, que recomendou seu uso para o diagnóstico inicial da TB e dos casos suspeitos de tuberculose multidrogarresistente (TB-MDR) em amostras de escarro^15^, e ampliou essa recomendação, em 2013, também para o diagnóstico de TB em amostras não respiratórias, ou seja, para a TB extrapulmonar^13^. Em função dessas recomendações, em 2014, foi disponibilizado um total de 3.269 aparelhos de GeneXpert aos setores públicos de 108 dos 145 países elegíveis para obtenção do equipamento^7^.

Frente ao exposto, esta revisão tem o objetivo de identificar e sintetizar o conhecimento científico a respeito da efetividade do teste GeneXpert no diagnóstico da TB pulmonar em pessoas que vivem com HIV/aids.

## MÉTODOS

Trata-se de uma revisão integrativa, a qual tem potencialidade de reunir informações sobre determinado tema, de modo a apresentar o estado da arte do objeto de estudo, direcionar definição de conceitos, identificar lacunas e rever teorias e análise metodológica, além de informar e auxiliar práticas e iniciativas em políticas relacionadas à questão^17^.

Para realizar esta revisão, foram adotadas as seguintes etapas: elaboração da pergunta do estudo; levantamento bibliográfico e seleção de estudos primários; extração das informações dos estudos selecionados; avaliação da qualidade metodológica dos estudos incluídos; síntese e discussão dos resultados encontrados. Dessa forma, a presente revisão foi norteada pela seguinte questão: Qual é a efetividade do teste GeneXpert no diagnóstico da TB pulmonar em pessoas que vivem com HIV/aids?

Tal questão permitiu a identificação de descritores, utilizando a estratégia PICO, proposta pela The Joanna Briggs Institute (2017)^18^, apresentada no Quadro 1.

**Quadro 1:** Descritores que derivam da pergunta do estudo, segundo a estratégia PICO.

Acrônimo	Definição	Descritores (vocabulário controlado em negrito)
P	População	Pessoas que vivem com **HIV/aids**
I	Intervenção	Realização do teste GeneXpert^®^ MTB/RIF^a^ em amostras de escarro
C	Controle ou comparador^b^	Comparação com outros testes diagnósticos
O	Desfecho *(outcome)*	Efetividade no diagnóstico da **tuberculose** pulmonar

^a^ Não foi encontrado vocabulário controlado para o termo em questão, mas o termo e suas derivações foram utilizados na busca.

^b^ Não foram utilizados como descritores na busca.

Fonte: Elaboração própria, 2019.

Os descritores em negrito citados no Quadro 1 fazem parte do vocabulário controlado consultado nos Descritores em Ciências da Saúde (DECS), por meio do qual também foi possível identificar os termos correspondentes nos idiomas espanhol e inglês. Para os termos em inglês, também foi consultado o *Medical Subject Headings* (MeSH). Por fim, também se buscou o vocabulário livre utilizado na escrita das publicações, por meio de buscas por sinônimos no DECS e no MeSH, bem como nas bases de dados selecionadas para o estudo.

O levantamento bibliográfico foi realizado em dezembro de 2019 utilizando o vocabulário encontrado (Quadro 2) e os operadores booleanos OR (entre as palavras de mesmo significado) e AND (entre os grupos de palavras de mesmo significado).

**Quadro 2:** Vocabulário utilizado na busca dos artigos para a realização da revisão integrativa sobre efetividade do teste GeneXpert® MTB/RIF no diagnóstico da tuberculose pulmonar em pessoas que vivem com HIV/aids.

Grupos de palavras	Vocabulário Controlado/vocabulário livre
G1	**HIV** OR Síndrome de Imunodeficiência Adquirida OR aids OR HIV OR Síndrome da Deficiência Imunológica Adquirida OR Síndrome de Deficiência Imunológica Adquirida OR Síndrome da Imunodeficiência Adquirida OR Vírus da Imunodeficiência Humana OR Vírus de Imunodeficiência Humana *(Português)* **VHI** OR “Acquired Immunodeficiency Syndrome” OR AIDS OR “Human Immunodeficiency Virus” OR “Immune Deficiency Syndrome” OR “Immuno Deficiency Syndrome” OR “Immuno-Deficiency Syndrome” OR “Immuno-Deficiency Syndromes” OR “Immunodeficiency Syndromes” OR “Immunologic Deficiency Syndrome” OR “Acquired Immunodeficiency”(*Inglês)* **SIDA** OR “Síndrome de Inmunodeficiencia Adquirida” OR “Síndrome de Deficiencia Inmunológica Adquirida” OR “Síndrome de la Inmunodeficiencia Adquirida” OR VIH OR “Virus de Inmunodeficiencia Humana” OR “Virus de la Inmunodeficiencia Humana” OR “Virus del SIDA” (*Espanhol)*
G2	**Tuberculose** OR TB *(Português)* **Tuberculosis** OR TB*(Inglês)* **Tuberculosis** OR TB *(Espanhol)*
G3	GeneXpert OR “teste rápido molecular” OR TRM OR “teste molecular rápido” *(Português)* Xpert OR GeneXpert OR Cepheid OR “rapid molecular test” *(Inglês)* “prueba rápida molecular” OR PRM OR “prueba molecular rápida”*(Espanhol)*

Fonte: Elaboração própria, 2019.

CINAHL: *Cumulative Index to Nursing and Allied Health Literature*; ASP: *Academic Search Premier*; SocINDEX: *bibliographic database for sociology research*; Lilacs: Literatura Latino-Americana e do Caribe em Ciências da Saúde; PubMed: *Public/Medline* ou *Publisher Medlin*; Scopus: *SciVerse Scopus* de propriedade da Elsevier; EMBASE: *Excerpta Medica dataBASE*.

O levantamento bibliográfico foi realizado em dezembro de 2019 nas seguintes bases de dados: *Excerpta Medica dataBASE* (EMBASE), *SciVerse Scopus* (Scopus) de propriedade da Elsevier, *Public/Medline* ou *Publisher Medlin* (PubMed), *Cumulative Index to Nursing and Allied Health Literature* (CINAHL), *Academic Search Premier* (ASP), *Bibliographic Database for Sociology Research* (SocINDEX) e Literatura Latino-Americana e do Caribe em Ciências da Saúde (Lilacs). Para a busca na Lilacs, utilizou-se o vocabulário nos três idiomas mencionados, e nas demais bases de dados, utilizou-se o vocabulário em inglês. É importante ressaltar que não foram utilizados limites de ano de publicação e nem de idioma no levantamento bibliográfico.

Tal busca resultou na identificação de 1.802 publicações, as quais foram exportadas para o aplicativo de revisão sistemática Rayyan QCRI da *Qatar Computing Research Institute*^19^, o qual identificou e possibilitou a exclusão de 597 publicações duplicadas. Dois revisores independentes fizeram julgamento da elegibilidade das publicações remanescentes por meio da leitura dos seus títulos e resumos. Na leitura integral dos artigos elegíveis (60), os seguintes critérios nortearam a inclusão de 19 estudos nesta revisão: estudos primários; ter pessoas com coinfecção TB-HIV como população de estudo; abordar o diagnóstico da TB pulmonar; e estudos que respondessem à questão norteadora da revisão. Cabe destacar ainda que os estudos considerados nesta revisão utilizaram somente a tecnologia GeneXpert produzida pela empresa Cepheid. Foram excluídos artigos que estudavam custo-efetividade, resumos publicados em anais e artigos que não comparavam o teste GeneXpert com outros testes diagnósticos (Figura).

**Figura:** Fluxograma da seleção dos artigos incluídos na revisão integrativa sobre efetividade do teste GeneXpert® MTB/RIF no diagnóstico da TB em pessoas que vivem com HIV/aids.

Fonte: Adaptado de Moher D et al. (2009)^20^.

Os dados dos artigos incluídos nesta revisão foram extraídos com a utilização de um instrumento específico baseado em Ursi (2005)^21^, o qual também permitiu uma síntese narrativa de seus resultados. Por fim, a qualidade metodológica dos artigos foi avaliada por meio do “*Checklist* para Estudos de Acurácia de Testes Diagnósticos” proposto pela *The Joanna Briggs Institute* (JBI) (https://joannabriggs.org/critical-appraisal-tools), que contempla dez itens de avaliação com respostas dicotômicas (sim/não), de tal forma que o item era considerado bem avaliado quando recebia a resposta “sim”. Dois itens do *checklist* não eram aplicáveis aos estudos encontrados e foram desconsiderados durante a avaliação.

## RESULTADOS

Dos 19 artigos incluídos nessa revisão, 18 (94,7%) foram publicados em língua inglesa^6,22^ e um (5,3%) em língua espanhola^39^, dos quais 15 (78,9%) são procedentes do Continente Africano^6,22,23,25^, dois (10,5%), do Continente Americano^37,39^, um (5,3%), do Continente Asiático^24^ e um (5,3%) estudo multicêntrico^35^envolve países da África, América, Europa e Ásia.

Os artigos foram publicados nos anos de 2011 (21,1%)^6,23,25,30^, 2012 (15,8%)^29,37,38^, 2014 (15,8%)^26,32,36^, 2015 (5,3%)^22^, 2016 (5,3%)^33^, 2017(15,8%)^24,28,34^ e 2018 (21,1%)^27,31,35,39^. Em relação ao tempo de investigação, quatro (21,1%) estudos tiveram duração de até 6 meses^22^, cinco (26,3%), de 6 meses até um ano^6,32,35,36,39^, oito (42,1%), de um a dois anos^25,26,28,29,31,33,37,38^ e dois (10,5%), de 2 a 3 anos^27,30^.

A síntese dos principais resultados sobre a efetividade do teste GeneXpert encontrados nos artigos incluídos na presente revisão são apresentados no Quadro 3. Onze estudos^6,23,25^ avaliaram a sensibilidade do teste, nove^6,23,25^ avaliaram a especificidade e oito, o valor preditivo positivo (VPP) e o valor preditivo negativo (VPN). Dos artigos incluídos no estudo, os exames de comparação utilizados para avaliar a efetividade do teste GeneXpert foram: 14 (73,6%) baciloscopia^6,23^, quatro (21,1%) cultura em meio sólido Lowenstein-Jensen (LJ)^32,34,35,39^, 13 (68,4%) cultura em meio líquido^6,22,23,25^, um (5,3%) Light Cycler Mycobacterium Detection (LCTB)^6^ e um (5,3%) MTBDRplus (Hain Lifescience)^6^. Três artigos^30,32,33^ avaliaram a efetividade do teste segundo os resultados da contagem de linfócitos T CD4+ e um artigo^33^ avaliou essa efetividade segundo o momento da coleta do escarro.

**Quadro 3:** Descrição dos artigos incluídos na revisão integrativa sobre efetividade do teste GeneXpert® MTB/RIF no diagnóstico da TB pulmonar em pessoas que vivem com HIV/aids.

Autores/Periódico/Ano/País	Delineamento do estudo	Objetivos	Total (n)	Instrumento de medida	Principais Resultados	Itens bem avaliados/ número de itens (Limitações)
Scott et al.^6^/ PLOS Medicine/ 2011/ Africa do Sul	Estudo prospectivo.	Avaliar o desempenho do teste Xpert MTB/RIF, do LightCycler Mycobacterium Detection (LCTB) e do MTBDRplus (Hain Lifescience) com a baciloscopia e a cultura líquida em uma amostra única de escarro.	311 indivíduos de uma população com alta prevalência de HIV.	GeneXpert MTB/RIF, microscopia de esfregaço pelo método Ziehl-Neelsen, cultura MGIT, MDRTBplus e LCTB.	GeneXpert – sensibilidade entre pessoas que vivem com HIV 84% (IC95% 69–93), especificidade 96% (IC95% 89–99), VPP 92% (IC95% 79–98), VPN 92% (IC95% 84–97). Baciloscopia – sensibilidade 54% (IC95% 38–69), especificidade 100% (IC95% 95–100), VPP 100% (IC95% 85–100), VPN 80% (IC95% 70-87). MDRTBplus – sensibilidade 70% (IC95% 54–83), especificidade 96% (IC95% 89–99), VPP 91% (IC95% 76–98), VPN 85% (IC95% 76–92). LCTB – sensibilidade 70% (IC95% 54–83), especificidade 98% (IC95% 93–100), VPP 97% (IC95% 83–100), VPN 86% (IC95% 77–92).	6/8(2N/A) - não é possível saber se os resultados do teste de referência (cultura de escarro) foram obtidos sem o conhecimento dos resultados do Xpert; - o estudo teve 22,6% de perda amostral.
Dagnra et al.^22^/ New Microbes and New Infections/ 2015/Togo	Não citou.	Determinar a prevalência de MDR entre pacientes HIV positivos e negativos previamente tratados e avaliar o desempenho do GeneXpert MTB/RIF na detecção de casos de MDR.	42 amostras de escarro.	GeneXpert MTB/RIF, cultura BACTEC MGIT 960 e microscopia com coloração Ziehl-Neelsen.	Apenas 31 (73,8%) dos 42 casos avaliados consentiram em fazer o teste de HIV. Desses, 07 (22,6%) eram HIV positivos. Todos os casos de coinfecção foram diagnosticados pelo GeneXpert e dois testaram positivo para resistência a rifampicina. Houve excelente concordância entre o teste de sensibilidade e o GeneXpert nas duas pessoas com MDR-TB.	6/8 (2 N/A) - processo de amostragem não elucidado e tamanho amostral reduzido, principalmente quanto ao número de pessoas com coinfecção; - não é possível saber se os resultados do teste de referência (cultura de escarro) foram obtidos sem o conhecimento dos resultados do GeneXpert.
Rachow et al.^23^/ PLOS ONE/ 2011/ Tanzânia	Estudo de validação clínica.	Comparar os resultados do GeneXpert à microscopia e à cultura nas amostras de escarro de pessoas com suspeitas de TB e infectadas ou não com HIV.	292 pacientes, e 876 amostras de escarro.	GeneXpert MTB/RIF, microscopia com coloração Ziehl-Neelsen e cultura em métodos LJ e BACTEC MGIT 960.	De 77 indivíduos em tratamento empírico para TB (baciloscopia e cultura negativa), 07 apresentaram positividade para o teste GeneXpert. Tais pessoas viviam com HIV. Sensibilidade do teste GeneXpert em pessoas que vivem com HIV: 82,0% (IC95% 68,6–91,4), da baciloscopia: 52,0% (IC95% 37,4–66,3) e da cultura (LJ): 68,0% (IC95% 53,3–80,5) e (MGIT): 76,0% (IC95% 61,8–86,9). Sensibilidade de ambas baciloscopia e cultura (LJ): 78,0% (IC95% 64,0–88,5). Especificidade para o teste GeneXpert foi de 98,0% (IC95% 89,4–100). Em relação aos outros testes, a especificidade foi de 100% (IC95% 92,9–100).	8/8 (2 N/A).
Sumangala et al.^24^/ Journal of International Medicine & Dentistry/ 2017/ Índia	Estudo transversal.	Avaliar a eficácia do Ensaio GeneXpert MTB/RIF no diagnóstico precoce de tuberculose em pessoas que vivem com HIV.	433 indivíduos e amostras de escarros.	GeneXpert MTB/RIF e microscopia fluorescente.	Das 433 amostras de escarro, 36 (8,3%) foram positivas para o GeneXpert e 28 (6,4%) amostras foram positivas para a microscopia fluorescente. Cinco casos de resistência à rifampicina foram detectados pelo GeneXpert.	5/8 (2N/A) - não é possível saber se os resultados do teste de referência (baciloscopia de escarro) foram obtidos sem o conhecimento dos resultados do Xpert e vice-versa; - utilizou a baciloscopia de escarro como método diagnóstico de referência, a qual apresenta limitações para classificar corretamente um caso da doença.
Lawn1et al.^25^/ PLOS Medicine/ 2011/ África do Sul	Estudo prospectivo.	Avaliar a precisão diagnóstica do teste GeneXpert MTB/RIF no diagnóstico da TB em pacientes com imunodeficiência avançada e sem terem iniciado a TARV.	445 indivíduos e 839 amostras de escarro.	GeneXpert MTB/RIF, microscopia, cultura líquida por BACTEC MGIT 960.	Dos 445 sujeitos participantes do estudo, 81 obtiveram resultado positivo para a cultura. GeneXpert – Sensibilidade 73,3% (IC95% 61,9–82,9); especificidade 99,2% (IC95% 97,7–99,8); VPP 94,8% (IC95% 85,6–98,9); VPN 94,8% (IC95% 92,1–96,8). O GeneXpert teve uma baixa taxa de falsos positivos e detectou todos os casos de TB positivos para ambos exames de baciloscopia e cultura. Baciloscopia – Sensibilidade 28,0% (IC95% 18,2–39,6); especificidade 100,0% (IC95% 98,9–100,0); VPP 100% (IC95% 83,9–100); VPN 87,3% (IC95% 83,7–90,3). O GeneXpert detectou 43,4% dos casos negativos à baciloscopia e positivos para a cultura em uma única amostra de escarro e 62,3% quando duas amostras de escarro foram analisadas. O teste identificou corretamente a resistência à rifampicina em quatro pacientes que tinham TB-MDR e apresentou três resultados falso positivos de resistência à droga.	8/8 (2N/A).
Balcha et al.^26^/ PLOS ONE / 2014/ Etiópia	Não citou.	Avaliar a prevalência de TB, o rendimento diagnóstico do GeneXpert MTB/RIF em comparação com a baciloscopia e a cultura e o impacto dos resultados do GeneXpert no tratamento clínico de adultos que vivem com HIV elegíveis para TARV.	812 indivíduos e 1.514 amostras de escarro.	GeneXpert MTB/RIF, microscopia com coloração Ziehl-Neelsen de esfregaço e cultura líquida.	Dos 137 pacientes com TB confirmados bacteriologicamente, 123 (89,8%) eram positivos para a cultura e 13 (10,2%) eram negativos. O teste GeneXpert foi positivo para 94 indivíduos, que também detectou 2 casos de resistência à rifampicina. A baciloscopia foi positiva para 31 pacientes. Em relação à microscopia de esfregaço, o GeneXpert aumentou a detecção de casos de TB em 47,4% (64 casos). Além disso, o GeneXpert detectou 14 (10,2%) casos de TB com cultura negativa ou contaminada. Entre 94 pacientes com amostras de escarro positivas para o GeneXpert, 30 (32%) eram positivas para baciloscopia. Para os 122 casos positivos para a cultura, 28 (23%) eram positivos para baciloscopia. Dos 104 casos de baciloscopia negativa, 54 (52%) foram positivos usando ambos GeneXpert e cultura, e 40 (38%) foram positivos usando apenas cultura. Todos os pacientes com baciloscopia positiva foram positivos para GeneXpert ou cultura. Além disso, 81 [66,4% (IC95% 57,2–74,6)] casos de TB positivos para a cultura foram positivos para o GeneXpert. GeneXpert – sensibilidade 96,4% (IC95% 79,7–99,9) e 57,4% (IC95% 46,8–67,5) entre casos de baciloscopia positiva e negativa, respectivamente; especificidade 98,4% (IC95% 97,0–99,2); VPP 88,0% (IC95% 79,2–93,6) e VPN 94,2% (IC95% 92,1–95,8). Baciloscopia – sensibilidade 23% (IC95% 16,0–31,7).	8/8 (2N/A).
Griesel et al.^27^/ Clinical Infectious Diseases/ 2018/ Africa do Sul	Estudo coorte prospectivo.	Desenvolver uma regra de previsão clínica para o diagnóstico da tuberculose e determinar a utilidade diagnóstica do ensaio GeneXpert MTB/RIF em pacientes infectados por HIV gravemente doentes.	484 indivíduos.	GeneXpert MTB/RIF, Cultura BACTEC MGIT 960 e microscopia de esfregaço por fluorescência.	Quanto à cultura de escarro, 53% (255/484) apresentaram resultado positivo. Dentre os participantes, 56 (11,6%) realizaram tratamento empírico com baciloscopia e GeneXpert negativos, dos quais 22 (43,1%) tinham resultado positivo para a cultura. GeneXpert – sensibilidade 86,3% (IC95% 81,5–90,3); especificidade 96,1% (IC95% 92,6–98,2); VPP 96,1% (IC95% 92,7–98,2); VPN 86,2% (IC95% 81,4–90,2); RVP 21,9 (IC95% 11,5–41,6); RVN 0,14 (IC95% 0,11–0,19). Baciloscopia – sensibilidade 57,0% (IC95% 50,7–63,2); especificidade 98,7% (IC95% 96,2–97,7); VPP 98.0% (IC95% 94.2–99.6); VPN 67.2% (IC95% 61.9–72.2); RVP 43,3 (IC95% 14,0–134,0); RVN 0,44 (IC95% 0,38–0,50).	7/8(2N/A) - não é possível saber se os resultados do teste de referência (cultura de escarro) foram obtidos sem o conhecimento dos resultados do Xpert.
Akanbi et al.^28^/ BMC Pulmonary Medicine/ 2017/Nigéria	Estudo coorte prospectivo.	Avaliar o impacto do GeneXpert MTB/RIF no diagnóstico, tempo de tratamento e resultado do tratamento entre pacientes com TB associada ao HIV na Nigéria.	310 indivíduos e 620 amostras de escarro.	GeneXpert MTB/RIF e microscopia de esfregaço.	A TB foi diagnosticada em 76 (24,5%) pacientes, dos quais 63 (82,9%) tiveram confirmação bacteriológica, 56 (73,7%) tiveram resultado positivo para o GeneXpert e 57 (75%) para baciloscopia. Na análise de concordância, 50 pacientes (65,8%) apresentaram resultado positivo para ambos exames, 6 (7,9%) eram positivos para o GeneXpert e negativos para a baciloscopia, 7 (9,2) negativos para o GeneXpert e positivos para a baciloscopia e 13 (17,1%) eram negativos para ambos exames.	5/8(N/A) - não é possível saber se os resultados do teste de referência (baciloscopia de escarro) foram obtidos sem o conhecimento dos resultados do Xpert e vice-versa; - utilizou a baciloscopia de escarro como método diagnóstico de comparação, a qual pode não classificar corretamente um caso da doença.
O’Grady et al.^29^/ Clinical Infectious Diseases/ 2012/ Zambia	Estudo descritivo, prospectivo.	Avaliar o desempenho do ensaio GeneXpert MTB/RIF para a detecção de tuberculose pulmonar e TB-MDR em pacientes adultos infectados pelo HIV e não infectados pelo HIV.	643 indivíduos.	GeneXpert MTB/RIF, microscopia de esfregaço, cultura BACTEC MGIT 960 e teste de sensibilidade a drogas MGIT.	GeneXpert em HIV positivos – sensibilidade 88,2% (IC95% 81,9–92,6); especificidade 95,1% (IC95% 91,5–97,3); VPP 92,2% (IC95% 86,5–95,7); VPN 92,5% (IC95% 88,4–95,3); sensibilidade quando a baciloscopia deu positiva: 96,5% (IC95% 89,3–99,1); sensibilidade quando a baciloscopia deu negativa: 78,9% (IC95% 67,8–87,1). GeneXpert em HIV negativos – sensibilidade 74,3% (IC95% 56,4–87,0); especificidade 96,3% (IC95% 91,7–98,5); VPP 81,3% (IC95% 63,0–92,1); VPN 94,5% (IC95% 89,5–97,3).	6/8(2N/A) - não é possível saber se os resultados do teste de referência (cultura de escarro) foram obtidos sem o conhecimento dos resultados do Xpert; - houve a exclusão de pacientes com amostras contaminadas e insuficientes e pacientes com cultura negativa em tratamento (o que representa uma perda de 33,4% dos casos elegíveis).
Theron et al.^30^/ American Journal of Respiratory and Critical Care Medicine/ 2011/ Africa do Sul	Não citou.	Validar o desempenho do teste GeneXpert MTB/RIF, usando um único cartucho e avaliar o impacto de alguns fatores no desempenho do teste.	480 amostras de escarro realizadas em uma população de alta prevalência de HIV.	GeneXpert MTB/RIF, microscopia de fluorescência e cultura BACTEC MGIT 960.	GeneXpert em pessoas com coinfecção – sensibilidade 69,6% (IC95% 55,2–80,1); sensibilidade em pessoas com baciloscopia negativa 47,3% (IC95% 29,2–67); especificidade 91,7% (IC95% 83,8–95,9); VPN 84,6% (IC95% 75,9–90,6); VPP 82,1% (IC95% 67,3–91). GeneXpert em pessoas não coinfectadas – sensibilidade 82,9% (IC95% 73,4–89,6); especificidade 95,6 (IC95% 91,8–97,7); VPN 93,3% (IC95% 89,1–96,0); VPP 88,3 (IC95% 79,3–93,4). Baciloscopia em pessoas com coinfecção – sensibilidade 50% (IC95% 36,1–63,9); especificidade 98,9% (IC95% 94,6–99,8). Em pacientes com HIV e contagem de CD4 < 200células/ml, a combinação da baciloscopia com o GeneXpert apresentou uma sensibilidade de 69,6% (IC95% 49,3–84,4) e a microscopia isolada de 39,1% (IC95% 22,2–59,2).	7/8(2N/A) - não é possível saber se os resultados do teste de referência (cultura de escarro) foram obtidos sem o conhecimento do resultado do Xpert.
Mbu et al.^31^/ PLOS ONE/ 2018/ Camarões	Não citou.	Avaliar a carga de TB entre as pessoas recém-diagnosticadas com HIV e comparar o desempenho do algoritmo de rastreamento clínico recomendado pela OMS e dos métodos de diagnóstico de TB nessa população.	940 indivíduos.	GeneXpert MTB/RIF, microscopia por fluorescência e cultura em meio LJ e BACTEC MGIT 960.	Nas 131 amostras com resultado positivo para cultura, foi realizado retrospectivamente o teste GeneXpert em 108 (82%) delas. GeneXpert – sensibilidade 68% (IC95% 58–76); sensibilidade em pessoas com baciloscopia positiva 97%; sensibilidade em pessoas com baciloscopia negativa 51%. Baciloscopia – sensibilidade da baciloscopia direta 24% (IC95% 18–32); sensibilidade da baciloscopia concentrada 35% (IC95% 28–44). Cultura: sensibilidade – sólida 81% (IC95% 73–87); líquida 98% (IC95% 94–99).	6/8(2N/A) - os resultados do teste de referência (cultura de escarro) foram obtidos antes do conhecimento dos resultados do Xpert; - o teste GeneXpert só foi realizado em amostras positivas para cultura, resultando em um perda amostral significativa.
Ssengooba et al.^32^/ PLOS ONE/ 2014/ Uganda	Estudo prospectivo.	Comparar o ganho diagnóstico de várias estratégias complementares do GeneXpert com o de uma estratégia de substituição do Xpert para o diagnóstico da TB pulmonar entre adultos que vivem com HIV.	424 indivíduos.	GeneXpert MTB/RIF, microscopia de esfregaço e cultura em meio sólido e líquido (MGIT e LJ).	123 (29,0%) participantes tiveram resultado positivo para cultura MGIT. Resistência à rifampicina – 04 positivas para GeneXpert e MGIT. GeneXpert – sensibilidade 76,4% (IC95% 67,9–83,6); sensibilidade entre os participantes com CD4 > 200 células/mm^3^ 91,7% (IC95% 73,0–98,9%); sensibilidade entre os participantes com CD4 ≤ 200 células/mm^3^ 73,2% (IC 63,2–81,7%). Quase não houve diferença na sensibilidade do teste entre os participantes com CD4 de 50 a 200 células/mm^3^ e participantes com CD4 < 50células/mm^3^ (68,9% vs 76,9%, p = 0,491). Cultura LJ – sensibilidade 81,3% (IC95% 73,2–87,7). Ziehl Neelsen – sensibilidade 31,7% (IC 23,6–40,7). Microscopia por fluorescência direta- sensibilidade 35,0% (IC95% 26,5–44,0). Microscopia fluorescente concentrada – sensibilidade 43,9% (IC95% 34,9–53,1).	6/8(2N/A) - amostragem não foi consecutiva nem aleatória (usou dados secundários); - não é possível saber se os resultados do teste de referência (cultura de escarro) foram obtidos sem o conhecimento dos resultados do Xpert.
Cavanaugh et al.^33^/ PLOS ONE/ 2016/ Quenia	Não citou.	Caracterizar o valor de vários testes de diagnósticos, incluindo o rendimento incremental do GeneXpert MTB/RIF e a cultura sobre a baciloscopia no escarro.	778 pessoas que vivem com HIV.	GeneXpert MTB/RIF, microscopia de esfregaço por Ziehl-Neelsen direto, microscopia de fluorescência concentrada, e cultura MGIT 960.	88 (11,3%) casos de TB foram confirmados. GeneXpert – detectou 43 casos (rendimento incremental = 24,4%). Nas amostras pontuais, identificou 48/84 casos (57,0%) enquanto o de amostras matinais identificou 50/76 (66,0%) casos. Dois testes GeneXpert detectaram 22/24 (92,0%) casos de TB com CD4 < 100células/μL e 30/45 (67,0%) dos casos com contagens de CD4 ≥ 100células/μl. O GeneXpert identificou 45 (28,0%; IC95% 16,6–39,3) pacientes com esfregaço negativo; identificou seis pacientes que não foram identificados por cultura. Microscopia – detectou 25 (33,6%) casos nas 76 amostras de escarro matinal, 24 (31,5%; IC95% 23,4–39,6) foram identificados por microscopia ZN e 29 (6,5% IC95% 2,8–10,2) por FD. Cultura líquida – identificou 10 (13,0%; IC95% 6,4–19,7) casos adicionais que não foram identificados por microscopia ou GeneXpert. No total, a cultura líquida e o GeneXpert realizados em uma única amostra matinal identificaram 60 (79,0%; IC95% 72,4–85) casos de TB pulmonar.	7/8(2N/A) - não é possível saber se os resultados do teste de referência (cultura de escarro) foram obtidos sem o conhecimento dos resultados do Xpert.
Mollel et al.^34^/ Tanzania Journal of Health Research/ 2017/ Tanzania	Estudo transversal.	Avaliar o desempenho do GeneXpert MTB/RIF no diagnóstico da TB em pacientes com coinfecção TB/HIV.	69 indivíduos.	GeneXpert MTB/RIF, microscopia e a cultura em método LJ.	O GeneXpert resultou em 09 resultados positivos para TB e 60 resultados negativos. Sensibilidade – 100% (IC95% 66,4–100); especificidade – 100% (IC95% 94–100); VPP 79,2% (IC95% 39–91,5); VPN 98,9% (IC95% 95–99,6). Microscopia – 05 positivos, 01 falso positivo, 59 negativos e 04 falso negativos. Sensibilidade – 55,6% (IC95% 21,4–86,3); especificidade – 98,3% (IC95% 91–100); VPP 75,6% (IC95% 28,9–95,9). VPN 96,0% (IC95% 92,0–98,0).	7/8(2N/A) - não é possível saber se os resultados do teste de referência (cultura de escarro) foram obtidos sem o conhecimento dos resultados do Xpert.
Dorman et al.^35^/ The Lancet Infectious Diseases/ 2018/ África do Sul, Uganda, Quênia, Índia, China, Geórgia, Bielorrússia e Brasil	Estudo prospectivo.	Comparar a acurácia diagnóstica do teste Xpert MTB/RIF Ultra com a do GeneXpert MTB/RIF para detecção de tuberculose com esfregaço negativo e resistência à rifampicina.	1.753 indivíduos adultos com sintomas de tuberculose pulmonar.	Xpert MTB/RIF Ultra, microscopia de esfregaço usando Ziehl-Neelsen e cultura MGIT BACTEC 960 e LJ.	As sensibilidades do Xpert Ultra e Xpert foram de 90% (IC95% 83–95) e 77% (IC95% 68–84), respectivamente, para os 115 participantes HIV positivos com escarro positivo para a cultura. As especificidades do Xpert Ultra e do Xpert para detecção foram respectivamente 96% e 98% em geral e 93% e 98% para pacientes com história prévia de tuberculose.	6/8(2N/A) - não é possível saber se os resultados do teste de referência (cultura de escarro) foram obtidos sem o conhecimento dos resultados do Xpert; - o estudo teve 26,0% de perda amostral.
Auld et al.^36^/ Public Health Action/ 2014/ Camboja	Não citou.	Descrever a implementação e utilização do teste GeneXpert MTB/RIF no diagnóstico da tuberculose entre pessoas que vivem com HIV/aids.	497 indivíduos.	GeneXpert MTB/RIF, microscopia de esfregaço e cultura.	Das 497 pessoas que viviam com HIV, 357 (72%) foram testadas com baciloscopia e 250 (50%) com Xpert; das quais 25 (10%) resultaram positivas para TB e nenhuma resistente à rifampicina. Apenas 22/357 (4%) realizaram cultura de escarro, sendo 04 (18%) positivas para *M. tuberculosis* e 06 (27%) para micobactérias não tuberculosas (MNT).	4/8(2N/A) - amostragem não foi consecutiva nem aleatória (usou dados secundários); - não é possível saber se os resultados dos testes de referência (baciloscopia ou cultura de escarro) foram obtidos sem o conhecimento dos resultados do Xpert e vice-versa; - em alguns casos, utilizou-se a baciloscopia de escarro como método diagnóstico de comparação, a qual pode não classificar corretamente um caso da doença; - o teste padrão de referência não foi o mesmo para todos os participantes.
Balcells et al.^37^/ The International Journal of Tuberculosis and Lung Disease / 2012/ Chile	Estudo transversal.	Avaliar a precisão diagnóstica do ensaio GeneXpert MTB/RIF na detecção da TB pulmonar em pacientes que vivem com HIV nos cuidados clínicos em comparação com a microscopia tradicional de esfregaço rápido e cultura.	160 indivíduos.	GeneXpert MTB/RIF, microscopia de esfregaço e culturas em meio LJ e MGIT BACTEC 960.	GeneXpert – sensibilidade 91,7% (IC95% 64,6–98,5); especificidade 99,3% (IC95% 96,3–99,9). VPP 91,7% (IC95% 64,6–98,5); VPN 99,3% (IC95% 96,3–99,9). Entre os 12 casos de TB com cultura confirmada, dois casos de resistência à rifampicina foram detectados pelo Xpert MTB/RIF (16,6%). Microscopia – sensibilidade 66,7% (IC95% 39,1–86,2); especificidade 98,6% (IC95% 95,2–99,6); VPP 80% (IC95% 49–94,3); VPN 97,3% (IC95% 93,3–99,3).	8/8(2N/A).
Lawn et al.^38^/ Clinical Infectious Diseases/ 2012/ Africa doSul	Não citou.	Relatar as características dos pacientes que vivem com HIV/aids com diagnóstico clínico de TB e resultado negativo para o GeneXpert MTB/RIF e relatar o status Xpert para subsequentes resultados pragmáticos e clínicos.	602 amostras de escarro.	GeneXpert MTB/RIF, microscopia de fluorescência e cultura líquida.	O *M. tuberculosis* foi cultivado em 01 amostra de 89 pacientes, o que representou uma prevalência de 17,0% (IC95% 13,9–20,5) de TB. Desses, 24 (27,0%) casos foram positivos para baciloscopia e 52 (58,4%) para o GeneXpert. Quando colhidas 02 amostras, a positividade para o GeneXpert foi de 64 (71,9%).	8/8(2N/A).
Cuervo et al.^39^/ Archivos Venezolanos de Farmacología y Terapéutica/ 2018/Cuba	Estudo retrospectivo.	Reafirmar o impacto-benefício do uso do GeneXpert MTB/RIF para o diagnóstico de tuberculose em pessoas que vivem com HIV/aids com suspeita de TB pulmonar.	152 amostras de escarros.	GeneXpert MTB/RIF, baciloscopia em meio Ziehl-Neelsen e cultura por LJ.	Das 152 amostras, o GeneXpert detectou 39 (25,65%) positivas de *Mycobacterium Tuberculosis*, sendo 36 (23,68%) não resistentes a rifampicina, 03 (2,00%) resistentes a rifampicina. A porcentagem de positividade da baciloscopia foi de 11,8% e da cultura por LJ foi de 21,7%. Ao compará-los, observa-se que a sensibilidade, especificidade e concordância deram acima de 90%, não obtendo diferenças estatisticamente significativo entre eles.	5/8(2N/A) - estudo é retrospectivo (possivelmente utilizou dados secundários); - informações sobre perdas e exclusões de pessoas da amostra não foram reveladas; - não é possível saber se os resultados do teste de referência (cultura de escarro) foram obtidos sem o conhecimento dos resultados do Xpert.

HIV: vírus da imunodeficiência humana; IC95%: intervalo de confiança de 95%; LJ: Löwenstein Jensen; MDR: multi-droga resistente; N/A: não se aplica; OMS: Organização Mundial da Saúde; RVP: razão de verossimilhança positivo; RVN: razão de verossimilhança negativo; TARV: terapia antirretroviral; TB: tuberculose; VPP: valor preditivo positivo; VPN: valor preditivo negativo.

Quanto à qualidade metodológica dos estudos, cinco estudos^23,25,26,37,38^contemplaram todos os itens considerados pela The Joanna Briggs Institute como indispensáveis a um bom estudo de avaliação da acurácia de testes diagnósticos. Apenas um (5,3%)^27^ artigo apresentou cálculo amostral.

## DISCUSSÃO

A epidemia e a expansão da infecção pelo HIV impactaram na incidência de TB^40^, cuja detecção precoce seguida de tratamento oportuno representam a chave para seu controle, de forma que o diagnóstico laboratorial representa um desafio para os serviços de saúde^39^. A população que vive com coinfecção por TB/HIV possui algumas características limitantes ao uso das ferramentas diagnósticas, uma vez que pode apresentar com mais facilidade esfregaço negativo, imunossupressão avançada que propicia atraso no diagnóstico e manifestação subclínica de sinais e sintomas característicos da TB^41^.

Em regiões endêmicas de TB/HIV que dispõem de escassos recursos, a microscopia de escarro geralmente é o único método disponível para o diagnóstico^4,32,34,42^. A utilização de apenas esta técnica para triagem de TB na população coinfectada com HIV restringe o seu potencial diagnóstico, uma vez que são necessários aproximadamente 10.000 organismos por mililitro no escarro para a execução do exame, quantidade incomum entre a população que vive com HIV^34^.

O teste GeneXpert é um método sensível, específico^43^, simples e inovador cujo objetivo, desde sua implantação, é detectar a presença de *M. tuberculosis* e a resistência à rifampicina em até 2 horas^6,22^. O método possui uma capacidade de triagem aumentada^43^, além de requerer concentrações mínimas bacilares nas amostras para a realização do exame^32^.

Em função dessas particularidades, questiona-se a relevância da baciloscopia para o diagnóstico da TB onde o GeneXpert está disponível, embora evidências encontradas mostrem que o uso concomitante de ambos os testes pode resultar em aumento da identificação de casos não detectados tanto por um exame quanto por outro^28,30^, principalmente em pessoas com contagem de linfócito T CD4+ < 200células/µl^30^.

Outros estudos não abordam a realização concomitante de ambos os exames, mas apontam que a utilização do GeneXpert aumentou substancialmente a detecção de casos de TB entre a população coinfectada com HIV^24,26,33,38,42^, sendo ela acentuada em amostras de escarro matinais e entre pessoas com menor contagem de linfócito T CD4+^33^.

A recomendação da OMS para o uso do GeneXpert ao invés da baciloscopia como teste inicial de escolha em indivíduos que vivem com HIV susceptíveis à TB^6^, corrobora com os achados dos estudos que mostraram que a sensibilidade do GeneXpert no diagnóstico da TB nas pessoas que vivem com HIV variou de 68%^31^ a 100%^34^ (média 81,1%), sendo maior que a sensibilidade da baciloscopia^6,23,25^, que variou de 23,0%^26^ a 66,7%^37^ (média 43,3%). Diante do exposto, a implementação do GeneXpert, um teste rápido e preciso em diferentes contextos clínicos, levou ao aumento significativo no diagnóstico da TB pulmonar, tanto quando usado como um teste de primeira escolha, quanto como complementar^45^.

É importante destacar que a sensibilidade é calculada tendo como referência a cultura de escarro, que é o exame considerado padrão-ouro para o diagnóstico da TB. Mesmo que esse exame seja referência, alguns estudos o comparam com o GeneXpert, apontando menor sensibilidade deste em relação à cultura pelos métodos LJ e BACTEC MGIT 960^31^, enquanto outro estudo aponta maior sensibilidade^23^ e outro, desempenho igual em relação ao método LJ^32^. Dois estudos trouxeram outros métodos diagnósticos que foram também comparados ao GeneXpert: um indicou sensibilidade semelhante dele e do teste LCTB^6^, e outro indicou sensibilidade menor quando comparado ao Xpert Ultra^35^. Dessa forma, cabe destacar a importância do uso do GeneXpert como teste de escolha para o diagnóstico da TB pulmonar em adultos que vivem com HIV, uma vez que apresenta efetividade semelhante e superior em relação à cultura e até mesmo a outros métodos moleculares, como o LCTB. No entanto, cabe destacar a importância de aprofundar os estudos em relação à comparação do exame com a cultura, visto que existem divergências em relação aos resultados sobre tal efetividade. Também merece cautela a indicação do Xpert Ultra como um método de escolha em substituição a todos os testes disponíveis, inclusive ao GeneXpert, uma vez que nesta revisão encontrou-se apenas um estudo que fez a comparação entre os dois testes^46^.

Ainda cabe destacar que a sensibilidade do teste GeneXpert nas pessoas que vivem com HIV foi maior do que nas pessoas HIV negativas^29^, contrapondo-se a outro estudo que afirmou que a sensibilidade nas pessoas com HIV foi menor^30^. A sensibilidade aumentada do teste em pacientes que vivem com HIV agrega vantagens importantes a seu uso nos serviços de saúde, como o aumento da taxa de detecção da TB microbiologicamente confirmada e a potencialidade de se instituir um tratamento rápido, com objetivo de reduzir a transmissão da TB^47^. Algumas características individuais, como apresentar baciloscopia negativa^26,29-31^ e contagem de linfócito T CD4+ ≤ 200 células/µl^32^, foram citadas como elementos que reduzem a sensibilidade do teste GeneXpert. Embora esses resultados sejam controversos em relação ao estudo de Cavanaugh et al. (2016)^33^, que mostra aumento da sensibilidade do teste em pessoas com contagem menor de linfócitos T CD4+, outro estudo mostra que o desempenho do GeneXpert em pessoas que vivem com HIV com imunossupressão avançada possui certas limitações^32^ que podem ser causadas pela menor concentração de micobactérias no escarro e pela apresentação oculta ou subclínica da doença^30^.

A especificidade do GeneXpert variou de 91,7%^30^ a 100%^34^ (média 95,6%), sendo que na comparação com a baciloscopia, alguns estudos revelam que ambos os exames apresentaram desempenho semelhante em relação ao aspecto mencionado^6,23,27,34,37^, e outros que o GeneXpert possui menor especificidade^25,30^. Em relação a outros exames diagnósticos, o estudo cita que a especificidade do teste GeneXpert é semelhante à da cultura^23^ e do LCTB^6^. A especificidade do teste em pessoas que vivem com HIV também foi semelhante em relação às pessoas não infectadas pelo vírus^29^. Frente ao exposto, verifica-se que a especificidade do teste GeneXpert é comparável aos demais testes diagnósticos.

O VPP, entendido como a probabilidade de se ter a doença quando o teste é positivo, variou de 79,2% a 96,1% (média 91%) para o GeneXpert em pessoas que vivem com HIV, sendo considerado semelhante à baciloscopia^6,25,27,34,37^ e ao LCTB^6^, bem como quando o teste é realizado em pessoas que não vivem com o vírus^30^, embora um dos estudos tenha apontado que o VPP foi menor nessas pessoas^29^. Já o VPN, entendido como a probabilidade de não se ter a doença quando o teste for negativo, variou de 84,6% a 99,3% (média 92,6%) para o GeneXpert em pessoas vivendo com HIV, sendo considerado semelhante^34,37^ ou maior^6,25,27^ em relação à baciloscopia e semelhante ao LCTB^6^ e em pessoas que não vivem com o vírus^29^, embora um dos estudos tenha apontado que o VPN é maior nessas pessoas^30^. Dessa forma, assim como na avaliação da especificidade, os resultados da efetividade do teste GeneXpert quanto ao VPP e ao VPN mostraram evidências de serem semelhantes aos demais testes de comparação.

Um estudo também apresentou a razão de verossimilhança do teste para o diagnóstico da TB em pessoas com coinfecção, a qual é calculada dividindo a probabilidade do resultado positivo (verossimilhança positiva) ou negativo (verossimilhança negativa) nas pessoas com a doença pela probabilidade do mesmo resultado nas pessoas sem a doença^49^. Em relação à razão de verossimilhança positiva, verificou-se não haver diferença entre o GeneXpert e a baciloscopia^27^. No entanto, a razão de verossimilhança negativa do GeneXpert apresentou-se menor e próxima a “zero”^27^, indicando ser melhor do que a baciloscopia.

A efetividade do teste na identificação de casos resistentes à rifampicina necessita ser melhor estudada, uma vez que um estudo^25^ mostrou que de sete casos identificados, três eram falso positivos; bem como pelo fato de outros estudos^6,22,24,32,37^ terem população muito reduzida de casos resistentes, limitando as conclusões de que o teste possui efetividade semelhante aos testes de sensibilidade^22,24,32,37^ e, inclusive, ao MDRTBplus^6^. Dessa forma, diagnosticar a resistência à rifampicina ainda permanece como um desafio para os serviços de saúde, pois possibilitaria a redução do tempo médio de detecção de casos resistentes a até dois dias^32^, o que seria um desempenho admirável quando comparado aos quase 40 dias do teste de sensibilidade/cultura convencional^25^. O diagnóstico imediato desses casos permitiria a redução substancial dos riscos de transmissão nosocomial do bacilo resistente e o seu tratamento específico e precoce, o que resultaria na redução das formas graves da TB, bem como na melhora no prognóstico dos indivíduos e na diminuição das perdas de seguimento clínico^25,39^.

Frente à efetividade do teste GeneXpert no diagnóstico da TB entre os indivíduos que vivem com HIV, torna-se indispensável sua expansão especialmente nos países com alta carga de coinfecção, uma vez que esse teste traria ganhos à detecção de casos ao substituir ou complementar o teste de baciloscopia e tornaria oportuno o tempo de tratamento^28^em relação à cultura, a qual possui limitações de custo e exigência técnica^32,33^. Cabe aqui destacar ainda que a precisão diagnóstica do GeneXpert, baseado em uma sensibilidade de 85% e uma especificidade de 97%, alcançaria um potencial de salvar mais de 400.000 vidas por ano^34^, mesmo que alguns casos realizem tratamento empírico, o qual seria diminuído em locais com a implementação do teste^27,38^.

Quanto à qualidade metodológica dos estudos, cinco estudos comtemplaram todos os itens considerados pela *The Joanna Briggs Institute* como indispensáveis a um bom estudo de avaliação da acurácia de testes diagnósticos^23,25,26,37,38^. Das limitações metodológicas encontradas nos estudos, ressaltam-se o tamanho amostral reduzido^22^; as perdas amostrais acima de 20%^6,29,35^; a falta de esclarecimento quanto ao processo amostral^39^ e ao processo de amostragem^22^; o uso de dados secundários^32,36,39^; a dúvida quanto a realização^6,22,27^ ou não realização^31^ de estudos cegos em relação aos resultados dos testes realizados; e a utilização de testes de referência diferentes para os participantes do estudo^36^ ou a utilização da baciloscopia como teste de referência^24,28,36^. Além disso, identifica-se um possível viés de seleção da população de um estudo, em que o teste GeneXpert só foi realizado em amostras positivas para a cultura^31^.

Entretanto, apesar de o teste ter maior custo do que a microscopia, os benefícios do tempo de diagnóstico e da indicação da resistência medicamentosa mostraram-se superiores por trazerem maiores sensibilidade e especificidade. Além disso, o teste estabelece o diagnóstico em uma proporção significativa de pacientes com esfregaço negativo e apresenta um valor de exclusão consolidado para TB-MDR^30,45^. Partindo dessa realidade, o GeneXpert pode ser utilizado como método de diagnóstico de primeira linha^24^.

Como limitações, esta revisão pode não ter incluído estudos relevantes que não foram indexados nas bases de dados pesquisadas, nem ter incluído a literatura cinzenta que poderia viabilizar a identificação de abordagem de outros tópicos/pontos relevantes sobre a temática.

Por meio deste estudo, é possível concluir que locais com alta incidência de HIV poderiam se beneficiar da implantação do teste GeneXpert, uma vez que sua efetividade no diagnóstico da TB pulmonar nessa população é expressiva quando comparada à da baciloscopia, teste que foi por muito tempo amplamente utilizado para a detecção dos casos. Quando comparado a outros exames, houve controvérsia em relação à efetividade do GeneXpert e dos diferentes tipos de cultura, efetividade semelhante à do LCTB e menor que a do Xpert Ultra. Além do aumento da detecção de casos de TB pulmonar entre as pessoas que vivem com HIV, o teste GeneXpert pode proporcionar benefícios como resultados mais rápidos do que os obtidos pela cultura (método mais sensível para confirmação da TB), culminando em detecção e tratamento oportunos, o que torna o GeneXpert uma ferramenta na luta contra a TB. Juntamente com a inovação do uso do GeneXpert, torna-se necessário o apoio e o fortalecimento dos serviços de saúde na aplicabilidade dessa técnica para o alcance das metas do desenvolvimento sustentável e da Estratégia *End TB*.
